# Stingy bots can improve human welfare in experimental sharing networks

**DOI:** 10.1038/s41598-023-44883-0

**Published:** 2023-10-20

**Authors:** Hirokazu Shirado, Yoyo Tsung-Yu Hou, Malte F. Jung

**Affiliations:** 1 School of Computer Science, Carnegie Mellon University, Pittsburgh, PA 15213 USA; 2 Department of Information Science, Cornell University, Ithaca, NY 14853 USA

**Keywords:** Social evolution, Information technology

## Abstract

Machines powered by artificial intelligence increasingly permeate social networks with control over resources. However, machine allocation behavior might offer little benefit to human welfare over networks when it ignores the specific network mechanism of social exchange. Here, we perform an online experiment involving simple networks of humans (496 participants in 120 networks) playing a resource-sharing game to which we sometimes add artificial agents (bots). The experiment examines two opposite policies of machine allocation behavior: *reciprocal bots*, which share all resources reciprocally; and *stingy bots*, which share no resources at all. We also manipulate the bot’s network position. We show that reciprocal bots make little changes in unequal resource distribution among people. On the other hand, stingy bots balance structural power and improve collective welfare in human groups when placed in a specific network position, although they bestow no wealth on people. Our findings highlight the need to incorporate the human nature of reciprocity and relational interdependence in designing machine behavior in sharing networks. Conscientious machines do not always work for human welfare, depending on the network structure where they interact.

## Introduction

Machines powered by artificial intelligence (AI) are increasingly incorporated into various economic and social interactions in human groups^1,2^, and it is also the case with resource sharing^3,4^. For instance, software agents exchange financial resources with human traders^5^; autonomous cars share roads with human drivers^6,7^; robots share tasks and roles with human teammates^8–10^; and chatbots are increasingly accepted to share time with humans to communicate and coordinate^11,12^. To improve human welfare in such hybrid systems, researchers and developers often unquestioningly seek human-like moral and social preferences in machines and AI^6,13^. However, what kind of individual behavior provides collective goods can depend on the network structure and dynamics where they interact with each other^14,15^. People develop social relations and power inequality through social exchange with limited resources, e.g. food, space, time, and explicit commitments^16,17^. Given the specific network mechanism of resource sharing, the machines rewarding immediate partners might neither rectify power disparities in a human group nor support welfare there^18^. Conversely, even making machines *deactivated* can become an option to improve people’s collective welfare when it balances transaction power among them^19^.

**Figure 1:**
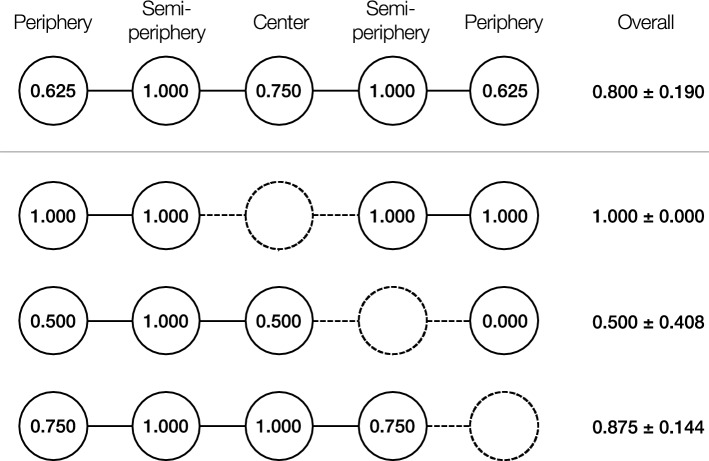
Sharing centrality in a five-node path graph. Numbers in nodes represents sharing centrality, i.e. the probability of having a mutual exchange. In social exchange, advantageous positions can differ from geodesic centrality. Moreover, deactivating a node (indicated with dashed outline) affects the structural power of all others including indirectly-connected nodes. The network effect varies across deactivated locations. The overall values are represented with average ± standard deviation.

We detail the theoretical prediction using a simple graph of five nodes to clarify the efficacy of machine behavior in this paper (Fig. 1), although the foundational theory can apply to any topology of networks where actors exchange limited resources. In the simple network, we name each node’s position based on its geodesic network location; the graph has two nodes of “periphery”, two nodes of “semi-periphery”, and one node of “center.” The geodesic locations are correlated with the measures of standard network centrality; e.g. eigenvector centrality, which computes the centrality for a node based on the centrality of its neighbors, is 0.577 for the “center” node, 0.500 for the “semi-periphery” nodes, and 0.289 for the “periphery” nodes (i.e. center > semi-periphery > periphery).

However, the “center” node is not the center of resource sharing. Following prior work^20^, we quantify such structural power in network exchange by the probability of having a mutual exchange, named “sharing centrality”. Sharing centrality captures the probability that a focal node will be engaged in a successful mutual selection with one of its neighbors. One way to understand how the geodesic position benefits a node is the following simple scenario with the model network (Fig. 1). Suppose the “center” node randomly selects one of its neighbors (i.e. one “semi-periphery” node) to allocate all its resources; then, if the “semi-periphery” node has also selected the “center” node as its unique partner, a symmetric and fully reciprocal relationship can be established, and these nodes will stop seeking exchange. Otherwise, the “center” node can proceed and select the other “semi-periphery” node as a potential exchange partner. This iterative process continues until a mutual selection is achieved for every possible pair of nodes. Each dyad has a different probability of mutual selection throughout this iterative process. Namely, the probability of mutual selection (by concurrent nomination in a repetitive process) is 0.375 for each “center”- “semi-periphery” edge and 0.625 for each “semi-periphery”- “periphery” edge. In total, this means that the “semi-periphery” nodes can find a full reciprocator with probability 1, while the probability is 0.750 for the “center” node and 0.625 for the “periphery” nodes (i.e. semi-periphery > center > periphery). The other centrality measures regarding network exchange show the same rank order^21^.

Prior work shows that the balance of exchange advantage among people can affect their welfare^22–24^. Note that the power inequality shown in Fig. 1 arises as a result of reciprocal altruism within local interactions^17,25,26^. Thus, even when an intervening machine behaves altruistically toward its human partners, it might not alter the status quo. In contrast, *deactivating* such a machine might make significant changes in the power distribution. A field experiment offers a suggestive finding that the people randomly selected to deactivate social media increase a sense of welfare with increased time meeting their friends and family members^19^, which indicates a negative association in time allocation. Moreover, whether it ameliorates or exacerbates the inequality depends on the network position of deactivation (shown in the lower half of Fig. 1). When the “center” actor is deactivated in the model network, the others equalize the sharing centrality. On the other hand, when one of the “semi-periphery” actors is deactivated, the structural power gap increases.

Here, we test the theoretical predictions with a virtual-lab experiment involving simple networks of humans playing an established economic game of resource sharing^21,23^, to which we sometimes add machine agents (“bots”). This experiment examines two opposite policies of machine allocation behavior: *reciprocal bots*, which share all resources reciprocally; and *stingy bots*, which share no resources at all. We evaluate the effect of each bot policy on collective welfare in human groups in the four specific dimensions: wealth, wealth inequality, satisfaction, and satisfaction inequality. Based on network exchange theory, we hypothesize that not reciprocal bots, but stingy bots can maintain and even improve collective welfare in human participants by facilitating reciprocity between them when located at a specific network position (Fig. 1). Specifically, we test the following hypotheses:

- **H1**: A stingy bot placed at the central node’s position will facilitate reciprocal exchanges between people and increase their group-wide satisfaction.
- **H2**: A stingy bot placed at a semi-peripheral node’s position will impede reciprocal exchanges between people and decrease their group-wide satisfaction.
- **H3**: A stingy bot placed at a peripheral node’s position will affect people’s reciprocal exchange but their group-wide satisfaction.
- **H4**: A reciprocal bot will make no significant improvements in sharing dynamics and group-wide satisfaction in humans, regardless of its geodesic position.

These hypotheses, especially H1 in contrast with H4, highlight peculiar network mechanisms in resource sharing. Stingy bots will undoubtedly be unsatisfying if we see a human–computer interaction alone. However, such a human–computer interaction is a piece of the complete picture, and the human (and the machine) also interact with other humans. From the network point of view, the interactions with machine partners can affect the interactions with human partners (and further). When machine agents aim for group-wide welfare, we might need to consider the chains of interactions in the overall effect of machine behavior. By testing the above paradoxical hypotheses, we illustrate the potential in the context of resource sharing, instead of advocating a specific policy in machine allocation behavior.

This research was approved by the Cornell University Committee of the Use of Human Subjects. All methods were performed in accordance with relevant guidelines and regulations. Informed consent was obtained from all participants. Our data include no identifying information of human participants. We conducted this experiment from May to June 2022. We preregistered the hypotheses and experimental procedure using AsPredicted^27^.

We recruited 496 human participants via the online labor market Amazon Mechanical Turk^28,29^ (see Table S1 for their demographics). We randomly assigned them to one of seven conditions in a series of 120 sessions (15–19 sessions per condition; Table S2). Participants were randomly assigned to a node’s position of the five-node path network and played a resource-sharing game for ten rounds (see “Methods”). We did not inform them when the game would end in order to prevent possible end-game effects^30^. Each player was given a certain amount of resource capacity, 30 units per round, which they could not spend on themselves. Instead, they could share this excess resource (which was useless to them) with their neighbors. In each round, players chose the number of resources they wanted to give to each of their neighbors (Fig. S1a). After all players in the network decided on their allocation, they were informed of their neighbors’ allocations to themselves. Then, they answered the question, “How happy are you with your neighbor(s) at this moment?” using a 5-level rating system with emojis (Fig. S1b). They continued the allocation decisions and feeling evaluation with the same network neighbors until they completed 10 rounds.

While making their decisions and evaluations, players were given information regarding their own resources and transactions. They were also informed about their own and neighbors’ total earnings until then (i.e. wealth). On the other hand, they were not informed about the entire network structure, the exchanges of their neighbors with the other players, the amount of their neighbors’ shareable resources, and their feeling evaluation. They could give their neighbors different amounts of the resource across the rounds, possibly recognizing past exchanges of their own or anticipating future ones, and they did not have to allocate their entire capacity. The non-allocated capacity neither carried over to the next round nor counted towards their wealth (hence, it was a *wasted* resource). Each player’s final wealth depended only on the units received from neighbors across the rounds, which were converted to actual monetary compensation at the end of the experiment ($1.00 = 200 units).

Following the 10-round game play, participants were asked about their satisfaction related to the game. They answered whether they agreed or disagreed, using a 7-level rating system, with the following 5 sentences: “In most ways, my outcome of the game was close to my ideal.” “My neighborhood situation in the game was excellent.” “I was satisfied with how the game went.” “I got a sufficient amount of resources from my neighbor(s) in the game.” “If I could play the game again, I would change almost nothing.” These questions were modified from an established satisfaction-with-life scale^31^ and used to evaluate the collective welfare of resource sharing in prior work^23^. With responses to the five items averaged, the resulting scale had high reliability (Cronbach’s alpha = 0.93), and the highest eigenvalue was 3.96. Therefore, we used the average score as a measure of the player’s satisfaction. We treated the satisfaction score as a more comprehensive evaluation of welfare than wealth because participants counted their eventual wealth in the post-game survey. We also confirmed that the satisfaction score was highly correlated with the average value of per-round feeling evaluation (by the five-level emojis) in individuals (Person’s correlation coefficient = 0.726; *p* < 0.001).

Within this basic setup, we introduced one bot into the network in exchange for one human player (no bots were placed in the control sessions). Participants were not informed that there was a bot in the game (except for supplementary sessions to examine the effect of bot identity; see below).

We manipulated the bot’s allocation policy as follows: In the “reciprocal bot” condition, the bots shared resources equally in the first round. After that, they allocated 30 units to each neighbor based on the neighbor’s percentage of total receiving units in the last round (see “Methods”). This bot policy yields a fair division of resources for their immediate neighbors^32,33^. In the “stingy bot” condition, the bots shared no resources at all with their neighbors. Thus, the bot’s neighbors received zero from it, regardless of how much they gave to the bot. This bot policy is seemingly antisocial and inefficient at both individual and collective levels. The bots solely wasted resources, reducing the wealth of the entire group.

Independent of the bots’ allocation policy, we also manipulate their network location based on geodesic centrality. A bot was assigned to the node of the “center,” “semi-periphery,” or “periphery” of a five-node path graph, as shown in Fig. 1.

In summary, we evaluated 7 conditions: 1 control condition not involving any bots; 6 treatment combinations of the allocation policy and network location of bots (2 types of allocation policy—reciprocal and stingy—with 3 types of network location —center, semi-periphery, and periphery). We preregistered the experiment to have 15–20 complete sessions for each condition^27^. As a result, we collected 120 completed sessions involving 496 participants (Table S2).

## Results

### Human wealth and satisfaction with bots

**Figure 2:**
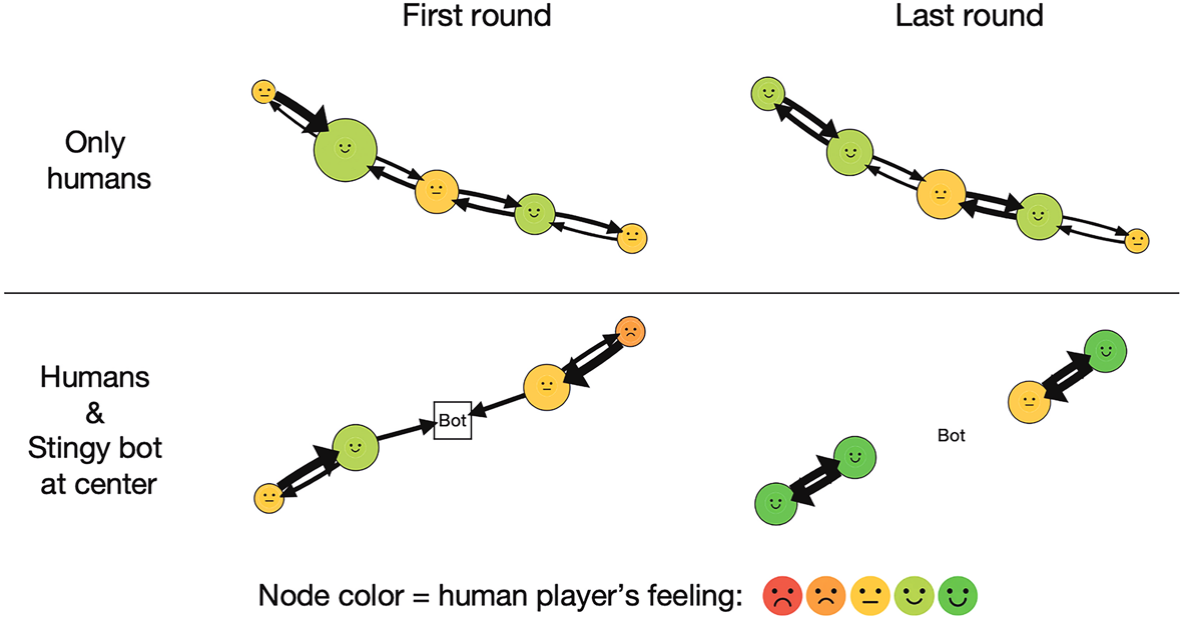
Structure and sharing snapshots at the first and last rounds. The snapshots are created from two actual sessions. Node position identifies each player. Node size is proportional to total earnings at the indicated round. Arrow size is proportional to shared resources from sender to receiver. Node color and label indicate player’s feeling after the sharing. Human players represent circle nodes, and stingy bots (i.e. the bots sharing nothing) represent square nodes.

Each player’s wealth and satisfaction depended on the behaviors of all players (including themselves and bots) in the resource sharing. Figure 2 shows two sample sessions’ structure and sharing snapshots at the first and final rounds. In the control session without bots, people exchanged their resources in the chains of interactions. Different network positions provided different magnitudes of benefits regarding wealth and satisfaction (respectively indicated as node size and color in Fig. 2). The structural inequality remained relatively steady across the rounds in the control session. On the other hand, players varied greatly in satisfaction over time in the session with a stingy bot located at the central node’s position. The treatment session had a similar imbalance in resource sharing in the first round. At the end of the game, however, human players held mutual exchanges with each other, whereas the bot did not receive any resources. In the session, the bot changed the sharing outcomes of not only the neighboring players but also the players with whom it did not interact directly.

**Figure 3:**
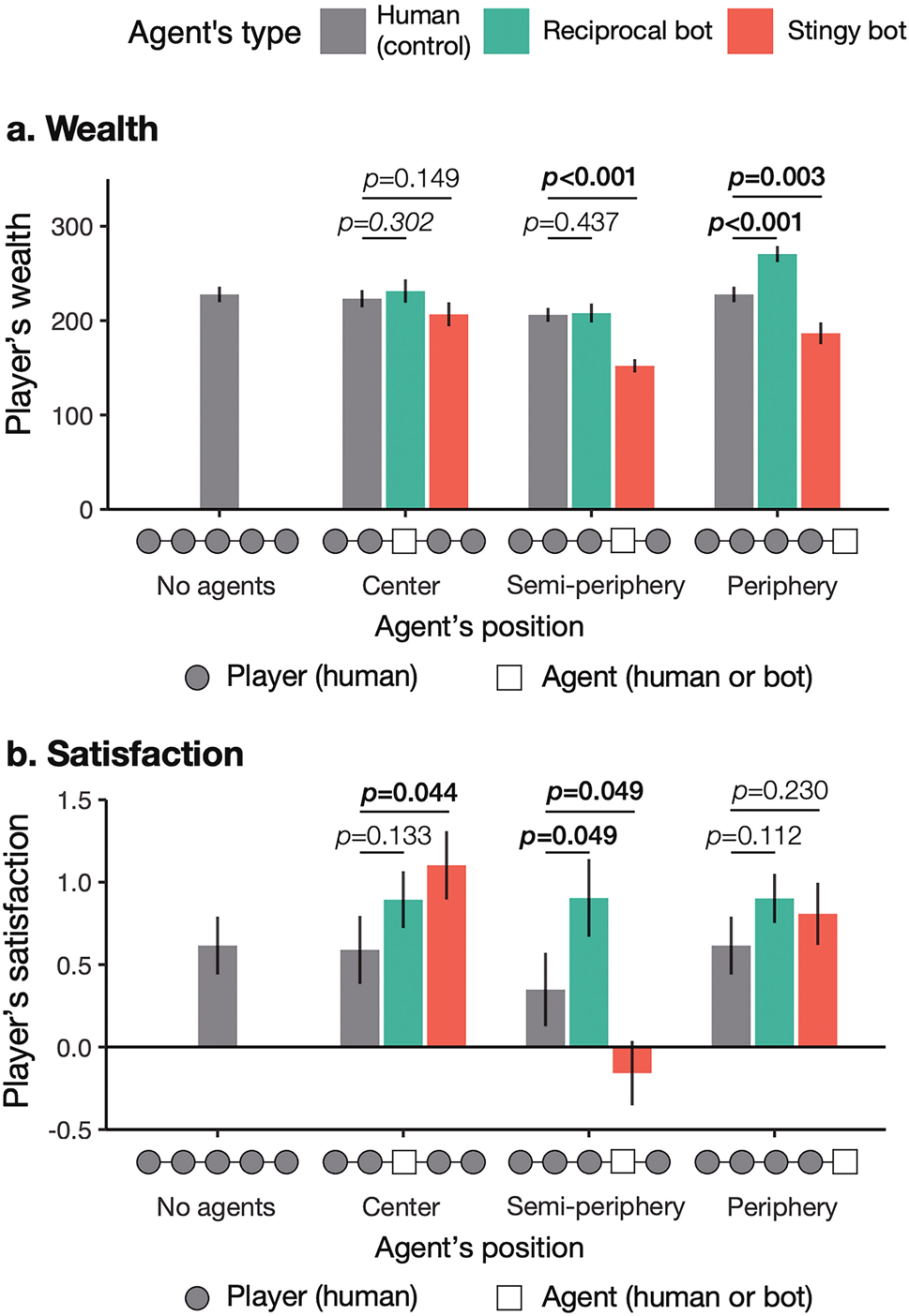
Average wealth and satisfaction across treatments. Error bars indicate standard error (*N*_*session*_ = 15–19; see Table S2). P values are calculated by one-sided *t* test; bold font indicates statistical significance at the 5% level. Only human players count in the analysis.

Figure 3 shows aggregated results of all the sessions regarding wealth and satisfaction across treatments. We did not count the bots’ wealth and satisfaction for the treatment sessions. Still, it could result in an inappropriate comparison with control sessions because wealth and satisfaction could vary by network position. Thus, the network average might increase or decrease simply by excluding the results of players located at a certain network position in the average calculation. To examine the behavioral effects of bots by comparison with the control sessions, we modified the network averages of the control sessions as if one human player per network were excluded as an agent in the calculation for each treatment (see “Methods”). For the statistical comparison, we used one-sided t-tests because, following the preregistration, we specifically tested the theory-driven directional hypotheses (**H1-4**) whether bots increase group-wide human welfare (or decrease it in the case of the stingy policy at a semi-periphery node’s position). We confirmed the *t* test results with a comprehensive analysis using regression models.

First, we found that the wealth effect of reciprocal bots is limited (Fig. 3a). Reciprocal bots always shared all resources, which many human players did not (61.7% of human players did not share all the resources across the rounds). Nevertheless, reciprocal bots did not increase people’s total wealth when placed in a central or semi-peripheral location. When an agent was located at the central node, the weighted average wealth of the control sessions is 223.2 and the average wealth of the reciprocal-bot sessions is 231.3 (*p* = 0.302 with one-sided t-test; Cohen’s *d* = 0.190); when placed in a semi-peripheral location, the weighted average wealth of the control sessions is 206.1 and the average wealth of the reciprocal-bot sessions is 208.1 (*p* = 0.437 with one-sided t-test; Cohen’s *d* = 0.058). This is because human players reduced their share when they had a reciprocal bot in their network at these positions (human players wasted 23.5% of resources in the control condition; 29.1% in the reciprocal-bot-at-center condition; 32.9% in the reciprocal-bot-at-semi-periphery condition; and 20.0% in the reciprocal-bot-at-periphery condition).

Figure 3a also shows the decrease in wealth by the stingy bot except when the bot was placed in the central location. Since stingy bots did not share any amount of resources during the game, human players should acquire less wealth (75 units per player on average) in the sessions with the stingy bot, compared with the human-only sessions. However, there is no significant downturn in players’ wealth from the control to the stingy-bot-at-center sessions (the weighted average wealth of the control sessions is 223.2; the average wealth of the stingy-bot sessions is 206.7; *p* = 0.149 with one-sided t-test; Cohen’s *d* = 0.347). This exception happened because people increased the share amount as the interaction evolved and covered the economic loss by the central stingy bot (Fig. S2).

This stingy-bot effect is highlighted when we see how people are satisfied with resource sharing including the bot (Fig. 3b). Stingy bots had larger effects on human players’ satisfaction than reciprocal bots, and whether the effects were positive or negative depended on their network position. Stingy bots, although doing nothing, *increased* people’s satisfaction when they were placed in the central location (the weighted average satisfaction of the control sessions is 0.590; the average satisfaction of the stingy-bot sessions is 1.102; *p* = 0.044 with one-sided t-test; Cohen’s *d* = 0.590; **H1**). On the other hand, stingy-bots decreased it when they were located at the semi-periphery (the weighted average satisfaction of the control sessions is 0.349; the average satisfaction of the stingy-bot sessions is -0.158; *p* = 0.049 with one-sided t-test; Cohen’s *d* = 0.590; **H2**) and did not change it when they were located at the periphery (**H3**). Reciprocal bots made no significant improvement in human satisfaction (**H4**) except when they were placed in a semi-peripheral location (the weighted average satisfaction of the control sessions is 0.349; the average satisfaction of the reciprocal-bot sessions is 0.904; *p* = 0.049 with one-sided t-test; Cohen’s *d* = 0.616). We found that reciprocal bots at a semi-periphery node’s position respond more fairly to the share amounts from local partners than humans at the same network position in the control sessions. This is the only result that disagrees with the pre-registered hypotheses.

The satisfaction result of the sessions with central stingy bots is especially striking because stingy bots that provided no wealth improved satisfaction. This finding suggests that human players experienced a sense of welfare besides economic quantity (i.e. wealth), and stingy bots facilitated the process from the central node’s position of the network without bestowing wealth and suppressed it from a semi-peripheral node’s position.

### Network heterogeneity of bot effects on wealth and satisfaction

**Figure 4:**
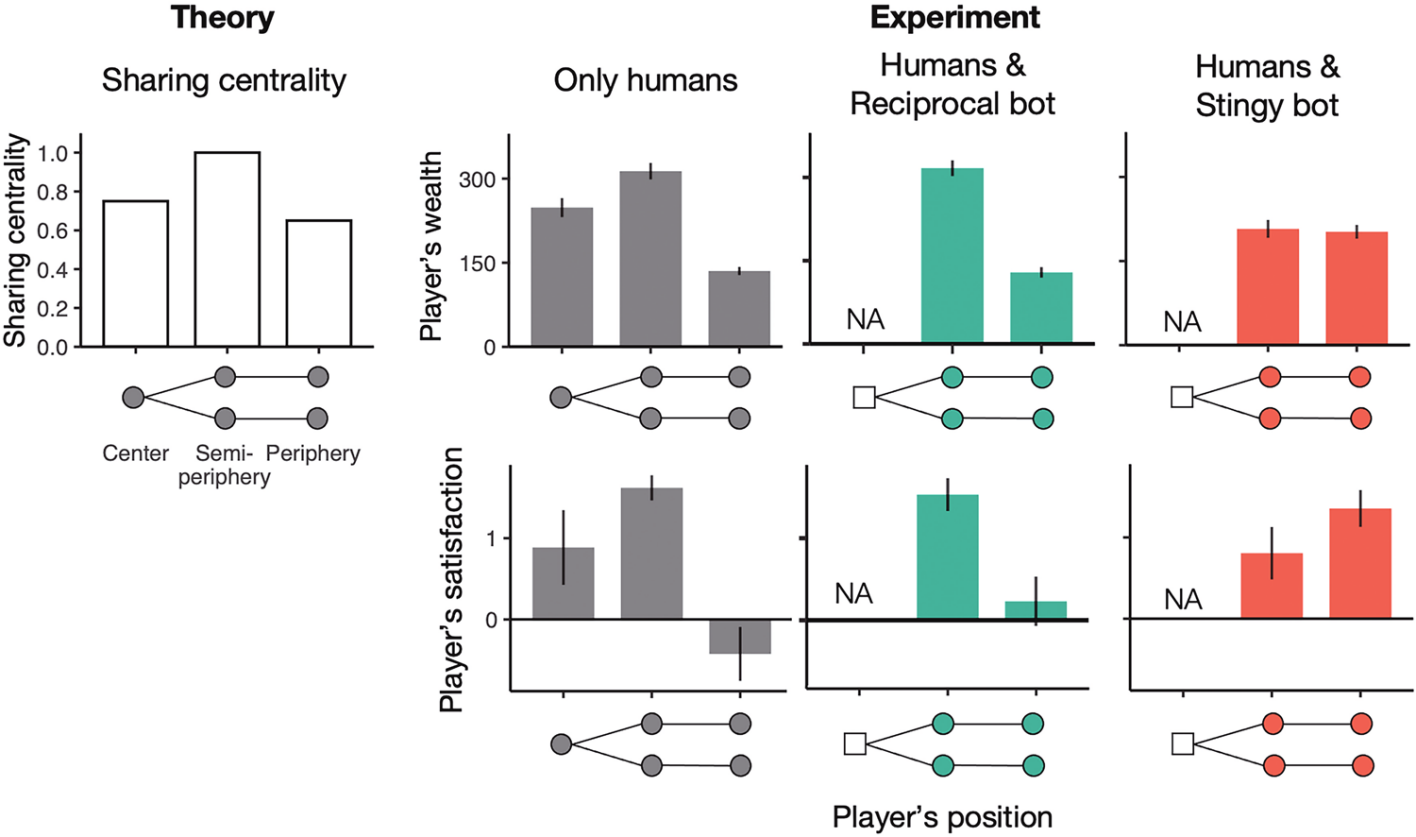
Network heterogeneity of wealth and satisfaction in the control and central-bot sessions. See Fig. S3 for the other bot positions. Error bars indicate standard error (*N*_*session*_ = 15–19; see Table S2).

Turning to the positional differences in the sharing outcomes, we find that economic benefits are highly correlated with structural power in the sharing network when it has only humans (Spearman’s rank correlation coefficient = 0.812 ± 0.035SE; *N*_*session*_ = 16). In the control sessions without bots, players’ sharing centrality significantly impacts their wealth (*p* < 0.001; Table 1). As the network exchange theory predicts, players earned the most when they were located at the semi-peripheral node’s positions; they earned the second most at the center position; they earned the least at the peripheral positions (Fig. 4).

**Table 1:** Results of the statistical analysis regarding the effect of sharing centrality with bot policies and positions on players’ wealth and satisfaction, estimated by regression models.

	Y = wealth				Y = satisfaction			
Dependent variables	Coeff	C.I. (2.5%, 97.5%)	P values		Coeff	C.I. (2.5%, 97.5%)	P values	
Intercept	− 138.6	(− 224.2, − 53.0)	0.002	**	− 3.566	(− 5.202, − 1.930)	0.000	***
Sharing centrality	458.6	(353.7, 563.4)	0.000	***	5.261	( 3.259, 7.264)	0.000	***
Bot treatments (ref. control)								
Reciprocal at center	− 45.3	(− 173.7, 83.0)	0.488		1.623	(− 0.823, 4.082)	0.193	
Reciprocal at semi-periphery	124.8	(− 12.3, 261.9)	0.074		3.866	( 1.246, 6.485)	0.004	**
Reciprocal at periphery	− 34.4	(− 164.4, 95.5)	0.603		1.052	(− 1.431, 3.535)	0.405	
Stingy at center	332.2	( 213.3, 451.0)	0.000	***	5.838	( 3.567, 8.110)	0.000	***
Stingy at semi-periphery	− 236.2	(− 375.5, − 116.9)	0.000	***	− 2.537	(− 5.008, − 0.066)	0.044	*
Stingy at periphery	270.6	( 137.1, 404.5)	0.000	***	6.076	( 3.526, 8.626)	0.000	***
Interaction effects								
Sharing centrality: reciprocal at center	42.1	(− 112.3, 196.5)	0.593		− 1.794	(− 4.744, 1.156)	0.233	
Sharing centrality: reciprocal at semi-periphery	− 161.3	(− 336.9, 14.2)	0.071		− 4.461	(− 7.816, − 1.107)	0.009	**
Sharing centrality: reciprocal at periphery	69.6	(− 84.9, 224.1)	0.377		− 1.195	(− 4.147, 1.757)	0.427	
Sharing centrality: stingy at center	− 444.6	(− 588.8, − 300.5)	0.000	***	− 6.728	(− 9.482 , − 3.974)	0.000	***
Sharing centrality: stingy at semi-periphery	262.3	( 97.4, 427.2)	0.002	**	2.645	(− 0.505, 5.795)	0.100	
Sharing centrality: stingy at periphery	− 389.6	(− 549.1, − 230.2)	0.000	***	− 7.327	(− 10.373, − 4.281)	0.000	***
Number of observations	464				464			
R-squared	0.508				0.222			
Adjusted R-squared	0.494				0.199			

The reference of bot treatments is the control without bots. Sharing centrality affects individual wealth and satisfaction. The interaction effects indicate how bots rectify or exacerbate the structural inequality represented by the effect of sharing centrality.

***p < 0.001; **p < 0.01; *p < 0.05.

We also find that reciprocal bots did not change the original network heterogeneity of wealth, regardless of the bots’ network position (Table 1). With reciprocal bots, semi-peripheral players still got rich and peripheral players still got poor (Fig. 4 and Fig. S3a). In contrast, stingy bots significantly altered the structural advantages and disadvantages of gaining wealth (*p* ≤ 0.001 for all the interaction coefficients with sharing centrality; Table 1), and their impact varied according to their network position. When a stingy bot played at the central node’s position of a network, humans’ wealth equalized between the semi-peripheral and peripheral players who had a major gap in original structural power (Fig. 3).

Players’ structural power of network exchange (i.e. sharing centrality) also affects their satisfaction by default (*p* < 0.001; Table 1). In the control sessions without bots, semi-peripheral players archived the highest satisfaction, then central players, and finally, peripheral players, which is in the same order as sharing centrality (Fig. 4). Bots changed the satisfaction distribution of human players in various ways depending on their allocation policy and network position (Table 1 and Fig. S3b). The improvements of structural inequality (i.e., the negative interaction effects) were significant when reciprocal bots were located at the semi-periphery (*p* = 0.009) and when stingy bots were located at the center and the periphery (*p* < 0.001 for both).

Of particular note is that, only with a stingy bot located at the “center,” all human players were satisfied with the game across their network positions (i.e. their satisfaction score was more than 0). While central stingy bots decreased the average satisfaction of players at the semi-peripheral node’s positions (from 1.619 to 0.806; *p* = 0.014; one-sided t-test), they significantly increased that at the peripheral node’s positions (from − 0.425 to 1.356; *p* < 0.001) (Fig. 4). The improvement for the people in the weak positions overwhelmed the suppression for those in the strong positions. As a result, group-wide satisfaction improved in the presence of a bot with inaction from the central node’s position (Fig. 3b).

### Stingy bots help humans reciprocate other’s share

We examined how players behaved from both their behavioral data during the game and subjective descriptions of their behavior in the post-game survey. In short, both analyses show that most human players allocate resources according to the norm of reciprocity.

**Figure 5:**
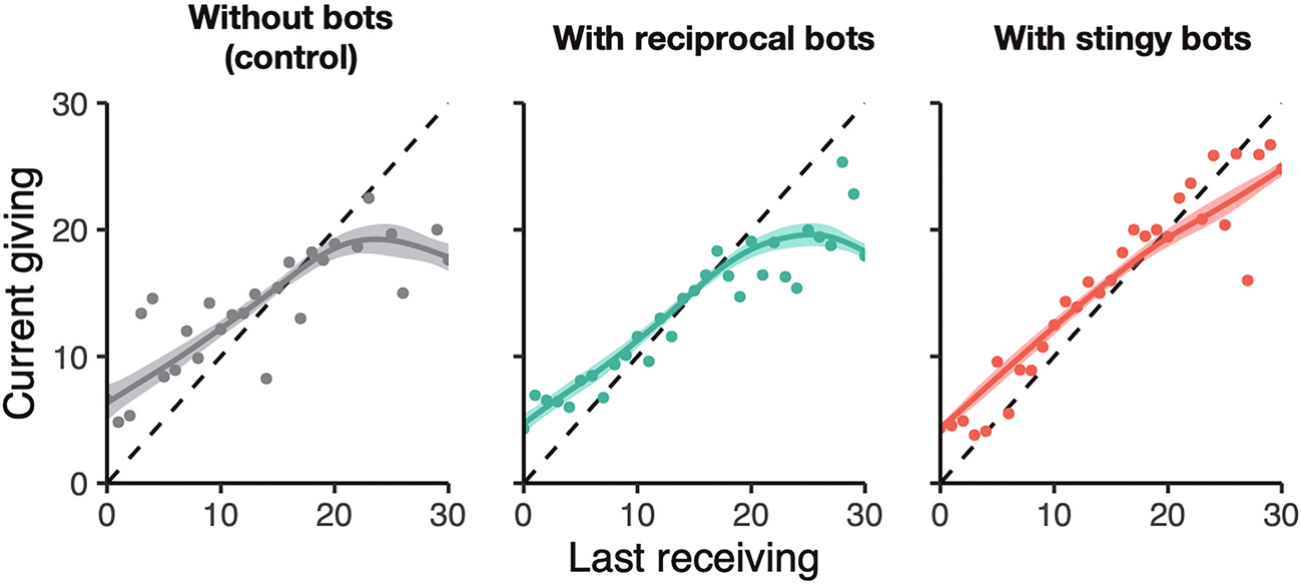
Human players reciprocate to neighbors and stingy bots help it. The graphs show how much a human player give their resources to a neighbor based on how much they received at the last round from the neighbor. Dots show the average of giving resources to a neighbor at round *t* by receiving resources from the neighbor at round *t*-1. Solid lines show smoothed conditional means estimated by generalized additive models. Shaded areas indicate standard error.

In the individual-level analysis of behavioral data, we focused on the level of reciprocity in their resource allocation across the treatments. Figure 5 shows how the player’s last-received resource amounts related to the next given ones for each neighbor. If a player strictly follows reciprocity, they will give the same amount of resources as they have received in the last round (indicated with the dashed lines in Fig. 5). The result shows that, in the control sessions, human players reciprocate to neighbors except when they have received more than 20 units in the last round. They often did not give back more than 20 units to a neighbor, even when they received that much. This deviation from reciprocity happened because they faced a structural constraint with limited resources. For example, if a player receives 20 units from two neighbors, the player cannot give 20 to both from the 30-unit capacity.

Although reciprocal bots did not change the situation, stingy bots did. In the session with stingy bots, human players reciprocated to neighbors, including when they received more than 20 units. The bots helped people overcome the structural constraint of reciprocal exchanges by sacrificing their own earning opportunities. Stingy bots did not increase human wealth. Nevertheless, when located at the central network position, stingy bots facilitated reciprocity not only with themselves directly, but also between human players indirectly. Facilitating group-wide reciprocity was likely to contribute to the increase in human satisfaction. We also confirmed the mechanism explanation with the results of post-game survey that asked participants about their satisfaction reasons (Fig. S4; see “Methods”).

### Robustness check with bot visibility

Considerable studies show that people behave differently with machine agents than other humans in economic games^34,35^. People often exploit cooperative approaches from machines and allocate fewer resources to them when they realize that their partners are machines. Identifying bots might counteract the behavioral treatment of bots in sharing networks.

Therefore, we conducted a separate experiment involving further 128 participants and a matched set of 32 networks (in addition to the 496 participants and 120 networks in the main experiment) and examined the impact of making the bots visible. In addition to the basic setting used before, human players were informed that they were interacting with bots and which nodes were played by bots by labeling the relevant nodes “bot” in their game view. As a result, we found no statistically significant difference with bot visibility. Group-wide wealth and satisfaction were statistically indistinguishable between the bot’s visible and invisible conditions (Fig. S5). In the reciprocal-bot sessions, the average wealth of the bot-invisible condition is 227.9 (15.4SE) and that of the bot-visible condition is 247.7 (14.7SE) (*p* = 0.355 with two-sided t-test; Cohen’s *d* = 0.173); the average satisfaction score of the bot-invisible condition is 0.894 (0.172SE) and that of the bot-visible condition is 0.950 (0.191SE) (*p* = 0.831 with two-sided t-test; Cohen’s *d* = 0.079). In the stingy-bot sessions, the average wealth of the bot-invisible condition is 204.8 (10.0SE) and that of the bot-visible condition is 209.2 (11.0SE) (*p* = 0.771 with two-sided t-test; Cohen’s *d* = 0.051); the average satisfaction score of the bot-invisible condition is 1.103 (0.208SE) and that of the bot-invisible condition is 1.051 (0.190SE) (*p* = 0.852 with two-sided t-test; Cohen’s *d* = 0.062). The bot’s behavioral effects on human players throughout the system are almost similar even when they identify their partners as bots in our experimental setup.

## Discussion

Our experiment supports the pre-registered hypotheses **H1-3** and partially supports **H4** about machine allocation behavior for human welfare developed from network exchange theory. It shows that reciprocal bots, which share reciprocally, do not improve collective welfare, except when they are placed in a semi-peripety location. The exception happens because reciprocal bots treat local partners more fairly than humans. In contrast, stingy bots, which share nothing at all, make a significant shift in people’s wealth and satisfaction. In particular, when stingy bots play at the central node’s position of the network, they improve people’s satisfaction by facilitating reciprocal transactions between them. Since stingy bots provide no wealth to people, this finding is evidence against a dyadic economic view of human-AI interaction. In assessing subjective welfare, people consider not only economic outcomes, but also social processes leading to the outcomes^23,24^. People can attain greater welfare even in a zero-sum game (where total wealth is on a plateau) when they are involved in reciprocal exchanges that are symmetric. This study uses a simple but illustrative AI to show the potential of designing machine agents for collective welfare to induce reciprocity in sharing networks.

One key difference from the standard notion of network effects is that exchanging limited resources provides negatively-connected networks, where one giving more to a partner has to give fewer to others^25,26^. In contrast, information diffusion and social contagion assume positively-connected networks, where one giving more to a partner can give more to others because information and other contagious social constructs are virtually unlimited in number^36–38^. In such networks, interventions are more straightforward; supporting specific individuals expects spillover effects on others^39^, and even without such effects, every increase in individual benefits adds up to the increase in collective benefits. Not all social interactions, however, are based on unlimited resources. In particular, people often develop social relations through social exchange with limited resources. Thus, when we design a system to facilitate social relations among people, we might need to consider the network mechanisms that differ from information diffusion^9,10^; otherwise, the system might yield unintended negative consequences^40^.

This study also suggests a complementary role of machine agents in facilitating human collectives^14,15^. In our experiment, reciprocal bots are less effective because their policy is similar to most human participants. Instead, stingy bots cause a significant shift in human groups, both positively and negatively, because they behave differently from typical humans (in fact, no human participants shared no resources in our experiment). In other words, stingy bots are insensitive to the norms of reciprocity, but humans are sensitive to them and treat the bots reciprocally (i.e., giving zero back to them). Thus, stingy bots can work as social catalysts for people when they are located at a specific network position. For exactly the same reason, however, introducing stingy bots is also quite risky; there are large negative consequences when they are misplaced (e.g. stingy bots at a semi-periphery node’s position of our study’s network). These findings suggest that to break up structural barriers, we might need to design machine behavior differently from human behavior because the barriers often come from current human practice, but it requires careful consideration of its position in an entire network^10^.

Our work involves human participants interacting online in a highly stylized way. Thus, we might miss features potentially relevant to resource sharing and subsequent welfare. For example, different people may have different shareable resources, and the differences can be associated with their network position^23,41^. Also, social equity might demand unequal resource allocations according to the need of each individual^42,43^. The type of resource sharing also might affect our findings. People might behave and feel differently with different types of resources (e.g. non-monetary resources). They might ensure welfare in a different configuration of its multiple dimensions in other contexts. In this study, we evaluate subjective satisfaction as a more holistic measure of welfare than objective monetary rewards because people count the rewards in their satisfaction assessment. However, regardless of whether individuals realize it, financial support could underpin long-term collective welfare. Finally, most real social networks are larger and more complex than the network we used^44,45^. In such a network, purely stingy bots might be hard to find an appropriate network position to work as much as our experiment shows (although it is not our research purpose). Developing an integrated algorithm of machine policies and network positioning is a promising next step to facilitate collective welfare, given more complex sharing networks. Our work provides theoretical guidelines with empirical evidence for the next step.

Although the results of laboratory experiments do not translate directly into the real world, the evidence presented here suggests further design space for machine allocation behavior by broadening the target scope to a network level. Network mechanisms provide the interdependence of social interactions, which is not always straightforward. Given that machines powered by artificial intelligence are increasingly incorporated into our economic and social life, they can leverage such network mechanisms to help people negotiate social impediments for collective welfare.

## Methods

### Participants

We conducted experiments from May to June 2022. A total of 496 unique participants were recruited from Amazon Mechanical Turk (MTruk) and completed our experiment. We decided the sample size based on prior work using the same experimental setting^23^. Participants could not join more than one session. We limited applicants to experienced workers on MTurk using the platform’s qualification system (1000 HITs completed with a 99% approval rate accomplished). Table S1 shows the participant demographics obtained with a free-response post-game survey. Before joining the game, all the participants passed an Internet speed check, human verification check, and a comprehension test about the experiment settings. Actual instructions are shown in the Supplementary Information.

### Experimental system

Experiments were implemented with the Breadboard platform (https://breadboard.yale.edu/). Participants interacted anonymously over the Internet using customized software playable in a browser window. To study the sharing dynamics and welfare in a network of people, we used a resource-sharing game based on the notion of a household WiFi sharing service (“WiFi sharing game”). This sharing game has been used to examine realistic sharing dynamics incorporating actual human interactions^21,23^, simulating real-world applications^46^. Although framed as a game involving the sharing of WiFi over geographic distance, the experiment setup has a number of generic features that are applicable to many settings where people have computer-supported social interactions and share resources^32^.

In the game, each player was given a certain amount of WiFi capacity, 30 units per round, which they could not spend on themselves (e.g. simulating an absence from their residence). Instead, they could share this excess resource (which was useless to them) with their neighbors. In each round, players chose the amount of resources they wanted to give to each of their neighbors (Fig. S1a). After all players in the network decided on their allocation, they were informed of their neighbors’ allocations to themselves. Then, they answered the question, “How happy are you with your neighbor(s) at this moment?” using a 5-level rating system with emojis (Fig. S1b). They continued the allocation decisions and feeling evaluation with the same network neighbors until they completed 10 rounds.

While making their decisions and evaluations, players were given information regarding their own resources and transactions. They were also informed about their own and neighbors’ total earnings until then (i.e. “wealth”). On the other hand, they were not informed about the entire network structure, the exchanges of their neighbors with the other players, the amount of their neighbors’ shareable resources, and their feeling evaluation. They could give their neighbors different amounts of the resource across the rounds, possibly recognizing past exchanges of their own or anticipating future ones, and they did not have to allocate their entire capacity. The non-allocated capacity neither carried over to the next round nor counted towards their wealth (hence, it was a *wasted* resource). Each player’s final wealth depended only on the units received from neighbors, which were converted to actual monetary compensation at the end of the experiment ($1.00 = 200 units). Thus, players were economically motivated to collect as much of this as possible over the course of the game.

### Experimental procedure

After the tutorial and screening process, participants were randomly assigned to one of seven conditions and a node of the five-node path network. They then played the WiFi-sharing game for 10 rounds (per session) without knowing when it would end. Following the 10-round game play, participants were asked about their satisfaction related to the game. They answered whether they agree or disagree, using a 7-level rating system, with the following 5 sentences: “In most ways, my outcome of the game was close to my ideal.” “My neighborhood situation in the game was excellent.” “I was satisfied with how the game went.” “I got a sufficient amount of resources from my neighbor(s) in the game.” “If I could play the game again, I would change almost nothing.” These questions were modified from an established satisfaction-with-life scale^31^ and used to evaluate the collective welfare of resource sharing in prior work^23^. Participants also described their gameplay to answer two open questions: “How did you decide your allocation to your neighbor(s) in the game?” “What made you happy or/and unhappy during the game?” Finally, they answered their socio-demographic status.

Participants received two fixed payments; the first corresponds to the traditional show-up fee ($2.00) and the second to the completion of the task ($2.00). The latter encouraged players to complete the game and post-game survey. Moreover, participants received a performance-based payment, which was proportional to the aggregate resources they received from all their neighbors throughout the game (i.e. *Wealth*_*i*_). The exchange rate of the bonus was $1.00 = 200 units.

Some participants were dropped during the game. When participants were inactive for 15 s in each decision-making, they got a warning about being dropped. When they remained inactive after 15 s, they were dropped. When at least one player was dropped halfway through the game, other players kept playing the game, but we did not use the data of all the players in the session rounds after the dropout. As a result, 36 of 156 sessions had at least one dropped player during the game (Table S2). We collected 120 completed sessions involving 496 participants. We used only the completed sessions except for the per-round analysis. We confirmed no statistically significant differences in the number of dropped sessions among treatments (*p* = 0.709; Fisher’s exact test).

After the gameplay, 32 of 496 participants did not complete the postgame survey (Table S2). In contrast to the gameplay, participants took the post-game survey individually. Thus, someone’s dropout did not affect other participants. We confirmed no statistically significant differences in the number of dropped participants in the post-game survey among treatments (*p* = 0.670; Fisher’s exact test).

### Measures

We evaluated the effects of experimental treatments compared with the control sessions without bots. We mainly focused on two variables as the game’s outcomes: human players’ wealth and satisfaction. We defined Player *i*’s wealth as

1 $${Wealth}_{i}=\sum_{t=1}^{10}\sum_{j\in {V}_{i}}{x}_{j\to i,t}.$$

Here *x*_*j*→*i,t*_ is the shared resources that Player *j* gives to Player *i* at round *t*, and *V*_*i*_ is the neighbors of Player *i*.

We measured each player’s satisfaction using the post-game survey of the five questionnaires. We transformed the 7-category answers (“strongly disagree,” “disagree,” “slightly disagree,” “neither agree nor disagree,” “slightly agree,” “agree,” and “strongly agree”) into numerical values from − 3 to 3, with higher values indicating higher satisfaction. With responses to the 5 items averaged, the resulting scale had high reliability (Cronbach’s alpha = 0.93), and the highest eigenvalue was 3.96 for the 5 items. Therefore, we used the average score as a measure of Player *i*’ satisfaction, *Satisfaction*_*i*_.

We then calculated network averages of wealth and satisfaction as collective outcomes. Network *k*’s average wealth and satisfaction were calculated as follow:

2 $${Wealth}_{k}=\frac{1}{{N}_{k}}\sum_{i=1}^{{N}_{k}}{Wealth}_{i},$$

3 $${Satisfaction}_{k}=\frac{1}{{N}_{k}}\sum_{i=1}^{{N}_{k}}{Satisfaction}_{i}.$$

Here *N*_*k*_ is the number of human players in Network *k*; *N*_*k*_ = 5 for the control sessions and *N*_*k*_ = 4 for the treatment sessions. We did not count the bots’ wealth and satisfaction for the treatment sessions. Still, it could result in an inappropriate comparison with control sessions because wealth and satisfaction could vary by network position. Thus, the network average can increase or decrease simply by excluding the results of players located at a certain network position in the average calculation. To examine the behavioral effects of bots by comparison with the control sessions, we modified network averages for the control sessions as if one human player per network were excluded as an agent in the calculation. Specifically, we introduced weights *w* in calculating the network averages of wealth and satisfaction for the control sessions:

4 $${Wealth}_{k}=\frac{1}{{\sum }_{i=1}^{{N}_{k}}{w}_{i}}\sum_{i=1}^{{N}_{k}}{w}_{i}*{Wealth}_{i},$$

5 $${Satisfaction}_{k}=\frac{1}{{\sum }_{i=1}^{{N}_{k}}{w}_{i}}\sum_{i=1}^{{N}_{k}}{w}_{i}*{Satisfaction}_{i}.$$

The weights *w* are based on what treatment to compare. When the control sessions are compared with the sessions with a central bot, *w* = 0 for human players located at the center; *w* = 1 otherwise. When compared with those with a semi-peripheral bot, *w* = 0.5 for human players located at the semi-periphery (because a bot was located at one of the two semi-peripheral node’s positions in a treatment network); *w* = 1 otherwise. Finally, when compared with those with a peripheral bot, *w* = 0.5 for human players located at the periphery; *w* = 1 otherwise.

Finally, we examined per-round outcomes across treatments. To discuss how economic transactions and satisfaction changed over time, we measured the following four parameters: per-round earnings, local Gini, local reciprocity, and feeling at each round. We modified the standard measurement of the Gini coefficient (which represents the wealth inequality within a social group)^47^ to the local wealth inequality that each player could recognize from their own and neighbors’ wealth. We also used a measurement of reciprocity in weighted networks^48^ to calculate each player’s local reciprocity in the sharing game.

We also calculated *Feeling*_*i,t*_ by transforming the 5-category answers to the question “How happy are you with your neighbor(s) at this moment?” into numerical values from − 2 to 2. We confirmed that the round average of *Feeling*_*i,t*_ was correlated with the overall satisfaction *Satisfaction*_*i*_ (measured from the answers to the five post-game questions) in individuals (Person’s correlation coefficient = 0.726; p < 0.001).

### Statistical analysis

We analyzed how individual wealth and satisfaction vary with sharing centrality and bot treatments using a linear regression model:

$$Y={\beta }_{0}+{\beta }_{1}{X}_{centrality}+{\beta }_{2}{X}_{bot}+{\beta }_{3}{X}_{centrality}{X}_{bot},$$

where *Y* is *Wealth*_*i*_ or *Satisfaction*_*i*_, *X*_*centrality*_ is a sharing-centrality value based on the player’s network position (i.e. the “center” node = 0.750; the “semi-periphery” nodes = 1.000; the “periphery” nodes = 0.625; Fig. 1), and *X*_*bot*_ is the vector of dummy variables indicating the bot treatment with reference to the control (i.e. reciprocal at center, reciprocal at semi-periphery, reciprocal at periphery, stingy at center, stingy at semi-periphery, and stingy at periphery). Table 1 shows the estimation results. The interaction effects ${\beta }_{3}$ indicate how bots rectify or exacerbate the structural inequality represented by sharing centrality.

### Supplementary Information

Supplementary Information.

## Data Availability

The datasets generated and analyzed during the current study are available in the Mendeley Data repository, https://data.mendeley.com/datasets/y88xdw2n97/1.
