# Supplementary material for: Stingy bots can improve human welfare in experimental sharing networks
Source: Sci Rep. 2023 Oct 20;13:17957. doi: 10.1038/s41598-023-44883-0 (PMC10589225; doi:10.1038/s41598-023-44883-0)
Supplement: Supplementary file 1 — Supplementary Information. [file 41598_2023_44883_MOESM1_ESM.pdf]

Supplementary Information for
Stingy Bots Can Improve Human Welfare in Experimental Sharing Networks

Hirokazu Shirado, Yoyo Tsung-Yu Hou, and Malte F. Jung

1. Supplementary figures and tables
2. Instruction and tutorials

1. Supplementary figures and tables

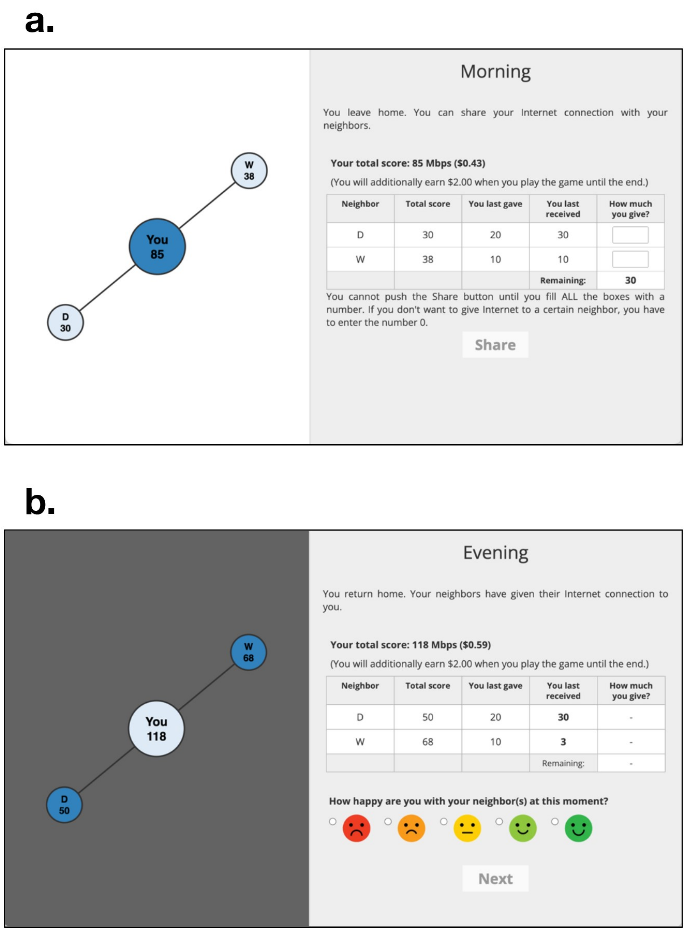

Figure S1. Sample player's view of WiFi sharing game. In each round, players decide on resource allocations to each neighbor based on the previous results. After being informed of their neighbors' share to themselves, they evaluated their feeling using a five-level rating system.

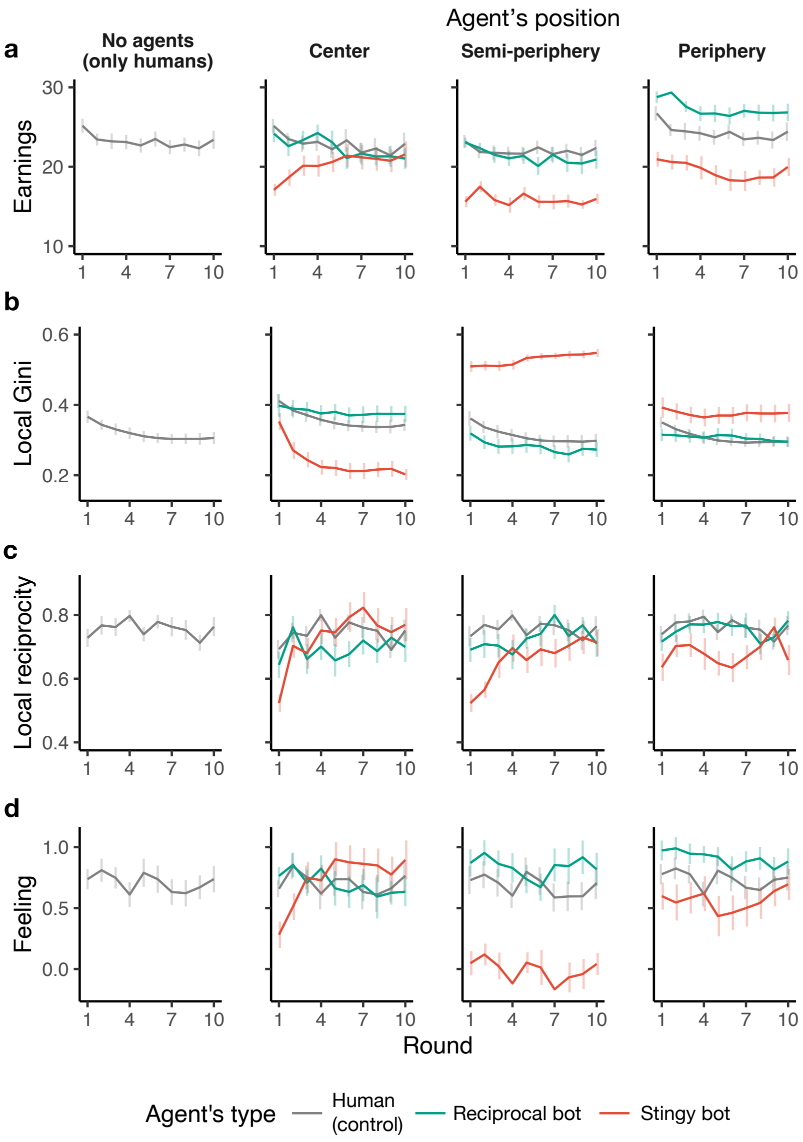

Figure S2. Per-round results across treatments regarding player's earnings, local Gini coefficient, local reciprocity, and feeling. All the economic outcomes are local parameters so that each player could recognize in their local network. The results do not include bots, but the calculation includes their allocation and outcome. Error bars indicate standard error (see Table S2 for $N_{session}$).

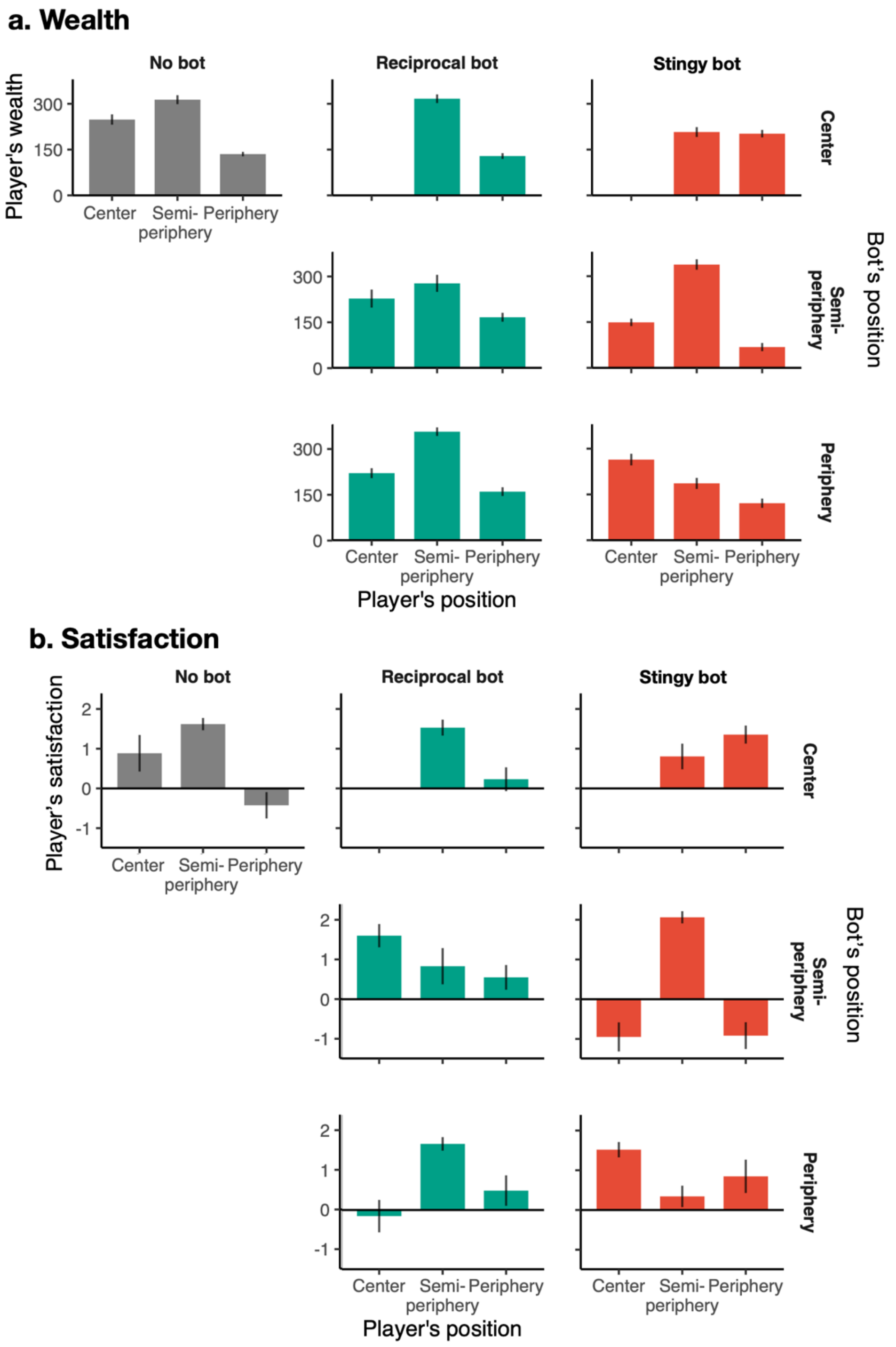

Figure S3. Network heterogeneity of wealth and satisfaction across treatments. Error bars indicate standard error.

Participants' sample answer	Category
Receiving solid amounts of internet from my neighbor made me happy. I wasn't very happy with the first two rounds, but then the neighbor started giving me more.	Wealth
When my sharing was reciprocated, I was happy. When it wasn't, it made me unhappy.	Reciprocity
What made me angrily unhappy would be my neighbors points keep going up but mine did not.	Social comparison
I was upset that I only got one neighbor and others seemed to have multiple.	Structural power
I was happy for it all; I was glad to share.	Altriuism
I am happy when the neighbor has a good score, I am unhappy when I have a good score.	Group welfare

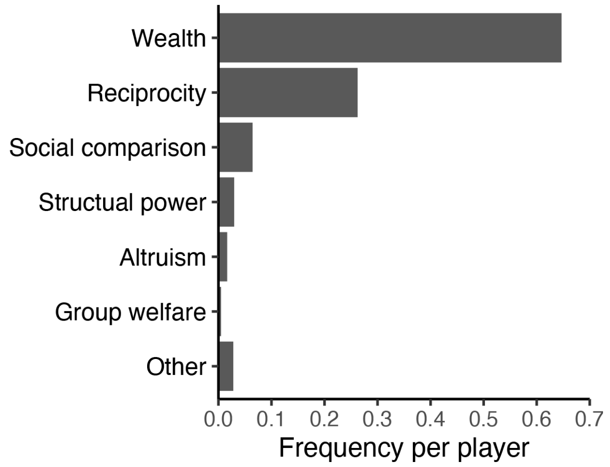

Figure S4. Categorization of player answers to the post-game question "What made you happy or/and unhappy during the game?" Multiple codes are given.

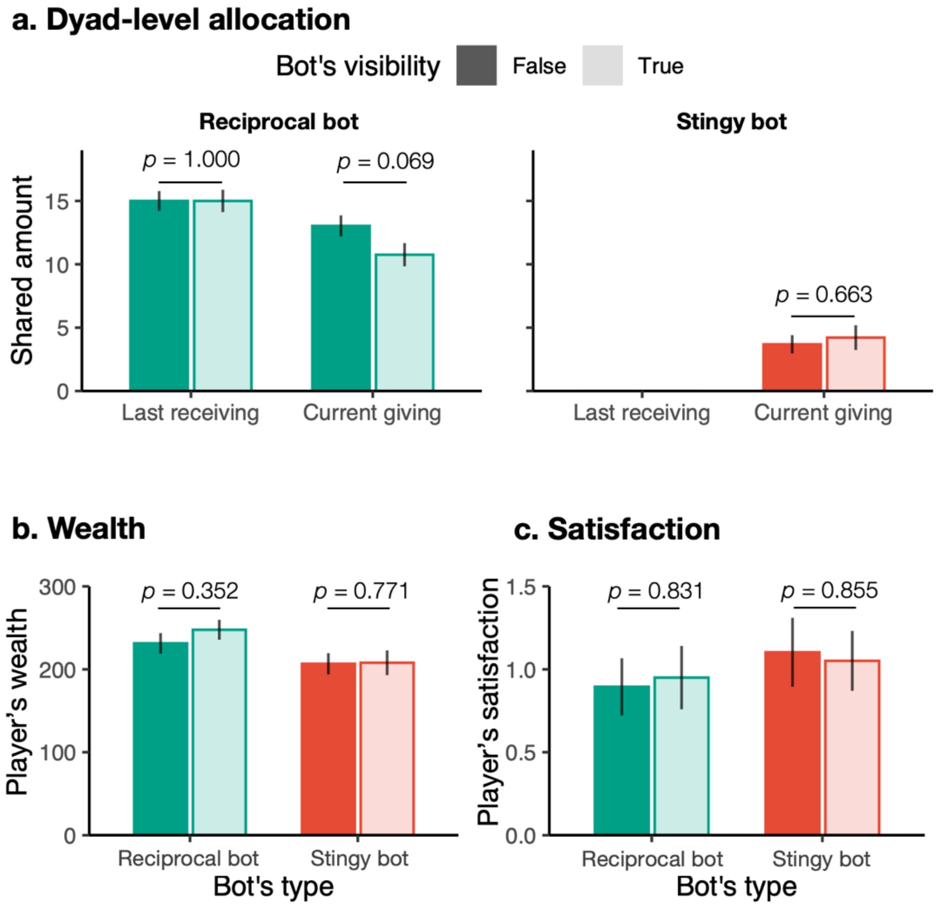

Figure S5. Differences in (a) dyad-level allocation, (b) wealth, and (c) satisfaction by bot visibility. P values are calculated by two-sided t tests.

Table S1. Demographics of experiment participants. The data were self-reported by the participants who completed the game session (N_{player} = 464).

Characteristics		Count	Percentage
Gender	Male	277	59.7%
	Female	183	39.4%
	Non-binary	1	0.2%
	No answer	3	0.6%
Age	20 - 29	97	20.9%
	30 - 39	217	46.8%
	40 - 49	76	16.4%
	50 - 59	52	11.2%
	≥ 60	19	4.1%
	No answer	3	0.6%
Nationality	American	350	75.4%
	Indian	82	17.7%
	Brazilian	12	2.6%
	Other	17	3.7%
	No answer	3	0.6%
Ethnicity	White, Caucasian, European; not Hispanic	300	64.7%
	Asian / Pacific Islander	108	23.3%
	Black / African american	19	4.1%
	Hipanic / Latino	25	5.4%
	American indican / Native american	1	0.2%
	Multiple ethnicity	6	1.3%
	No answer	5	1.1%
Education	High school or less	35	7.5%
	Some college (1-3 years)	77	16.6%
	Bachelr's degree	271	58.4%
	Graduate degree	76	16.4%
	No answer	5	1.1%
Annual income, $	0 - 20,000	135	29.1%
	20,000 - 34,999	84	18.1%
	35,000 - 49,000	78	16.8%
	50,000 - 74,900	88	19.0%
	75,000 - 99,999	46	9.9%
	≥ 100,000	21	4.5%
	No answer	12	2.6%

Table S2. Number of completed and dropped sessions across treatments. Some players dropped during a game session. In that case, the rest of session is excluded in the analysis. We collected 120 completed sessions involving 496 participants. After the session completed, *N*=32 players did not complete the post-game survey. There are no statistical differences in both session and post-game survey dropouts across treatments.

Treatment	Number of game sessions (N_{session})			Number of players per session	Number of post-session-survey players (N_{player})		
	All	Dropped	Completed		All	Dropped	Completed
No bots (only humans)	19	3	16	5	80	3	77
Reciprocal bot at Center	19	4	15	4	60	5	55
Recipocal bot at Semi-periphery	23	8	15	4	60	4	56
Reciprocal bot at Periphery	27	8	19	4	76	3	73
Stingy bot at Center	24	5	19	4	76	4	72
Stingy bot at Semi-periphery	21	3	18	4	72	6	66
Stingy bot at Periphery	23	5	18	4	72	7	65
Total	156	36	120	-	496	32	464

2. Instruction and tutorials

Below are screenshots for the initial description of the tutorial and the confirmation tests. We also show example screenshots of a real game.

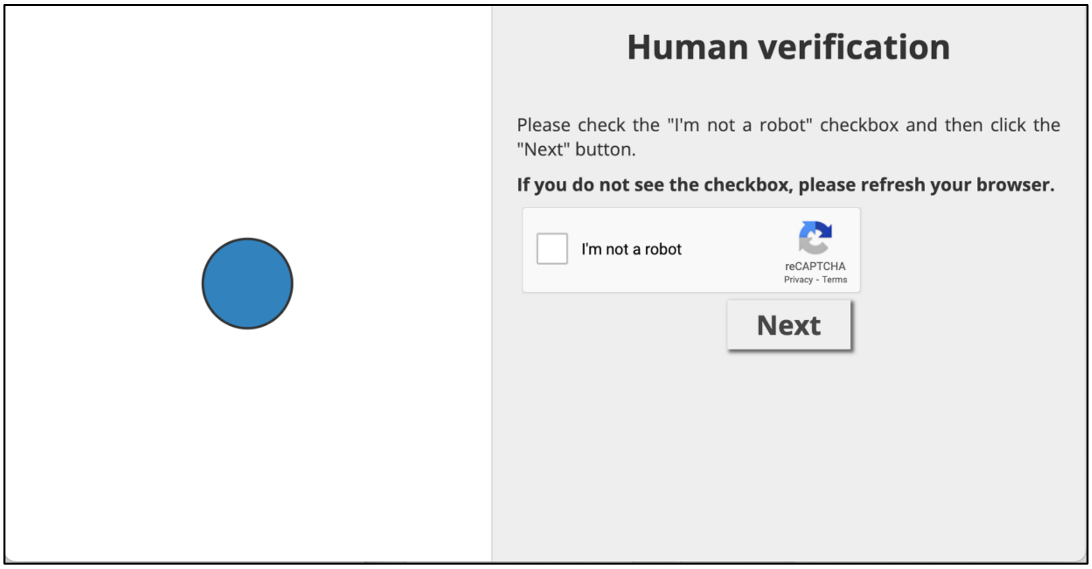

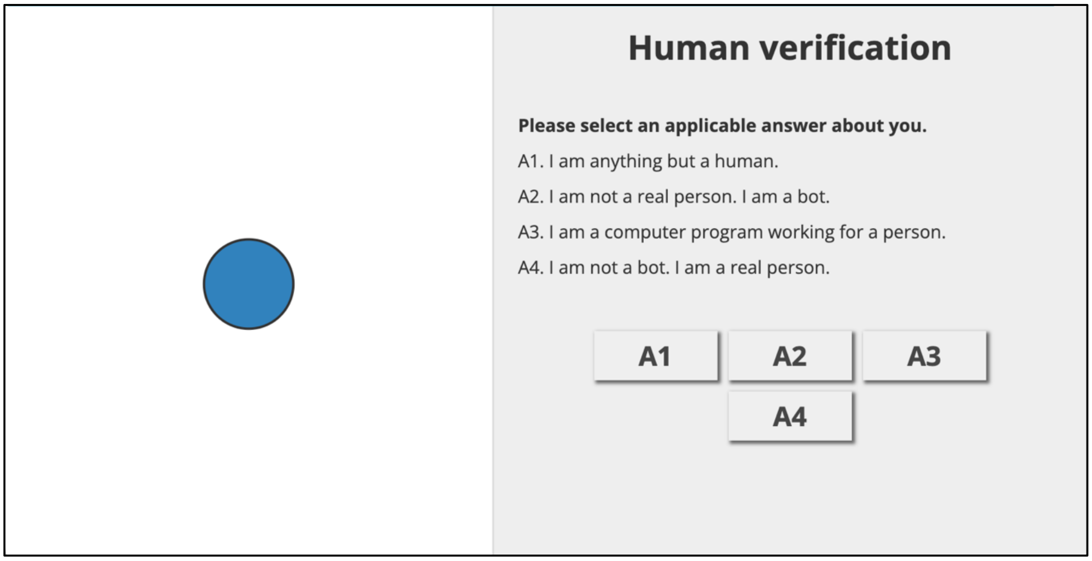

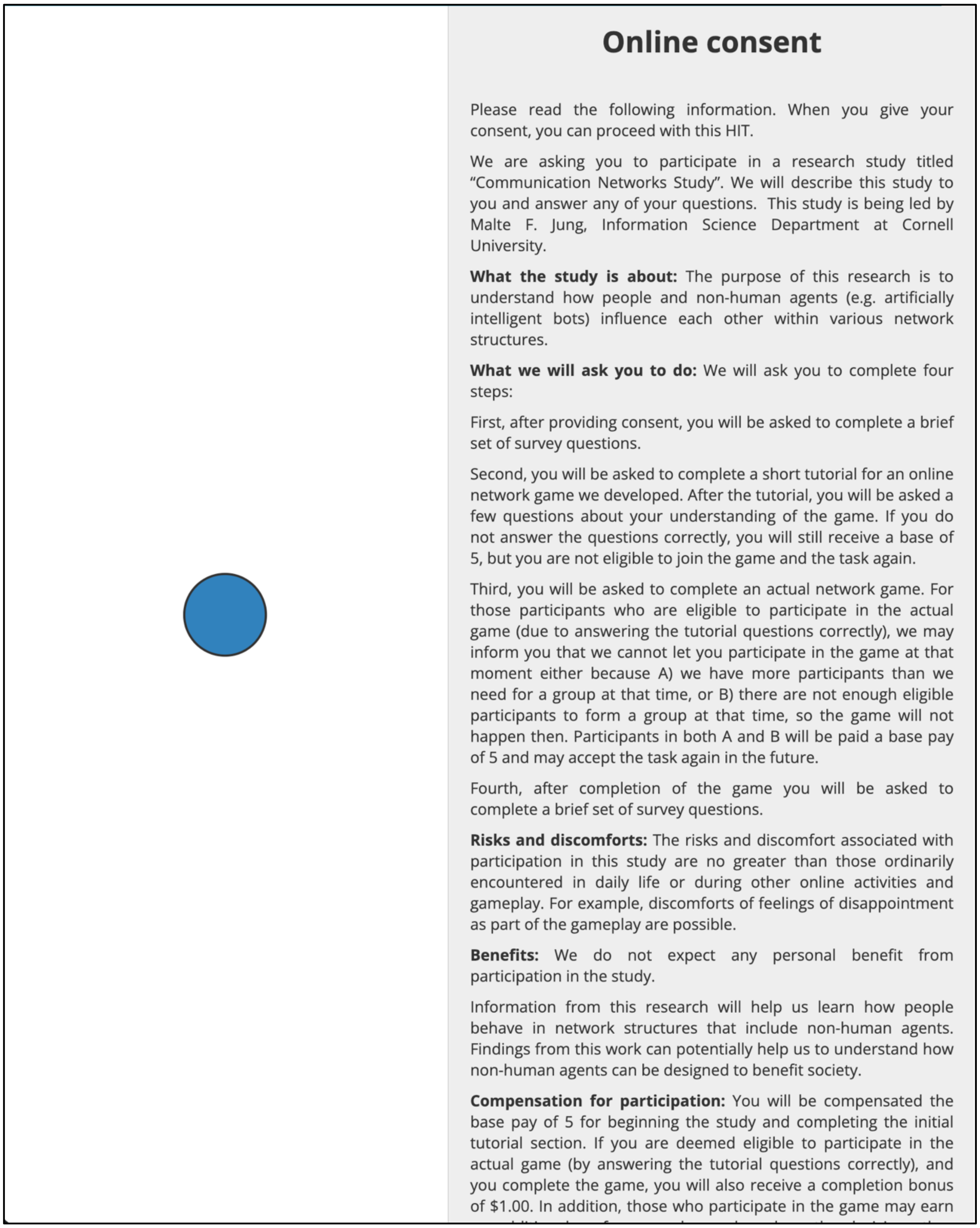

Online consent

Please read the following information. When you give your consent, you can proceed with this HIT.

We are asking you to participate in a research study titled "Communication Networks Study". We will describe this study to you and answer any of your questions. This study is being led by Malte F. Jung, Information Science Department at Cornell University.

What the study is about: The purpose of this research is to understand how people and non-human agents (e.g. artificially intelligent bots) influence each other within various network structures.

What we will ask you to do: We will ask you to complete four steps:

First, after providing consent, you will be asked to complete a brief set of survey questions.

Second, you will be asked to complete a short tutorial for an online network game we developed. After the tutorial, you will be asked a few questions about your understanding of the game. If you do not answer the questions correctly, you will still receive a base of 5, but you are not eligible to join the game and the task again.

Third, you will be asked to complete an actual network game. For those participants who are eligible to participate in the actual game (due to answering the tutorial questions correctly), we may inform you that we cannot let you participate in the game at that moment either because A) we have more participants than we need for a group at that time, or B) there are not enough eligible participants to form a group at that time, so the game will not happen then. Participants in both A and B will be paid a base pay of 5 and may accept the task again in the future.

Fourth, after completion of the game you will be asked to complete a brief set of survey questions.

Risks and discomforts: The risks and discomfort associated with participation in this study are no greater than those ordinarily encountered in daily life or during other online activities and gameplay. For example, discomforts of feelings of disappointment as part of the gameplay are possible.

Benefits: We do not expect any personal benefit from participation in the study.

Information from this research will help us learn how people behave in network structures that include non-human agents. Findings from this work can potentially help us to understand how non-human agents can be designed to benefit society.

Compensation for participation: You will be compensated the base pay of 5 for beginning the study and completing the initial tutorial section. If you are deemed eligible to participate in the actual game (by answering the tutorial questions correctly), and you complete the game, you will also receive a completion bonus of $1.00. In addition, those who participate in the game may earn [illegible]

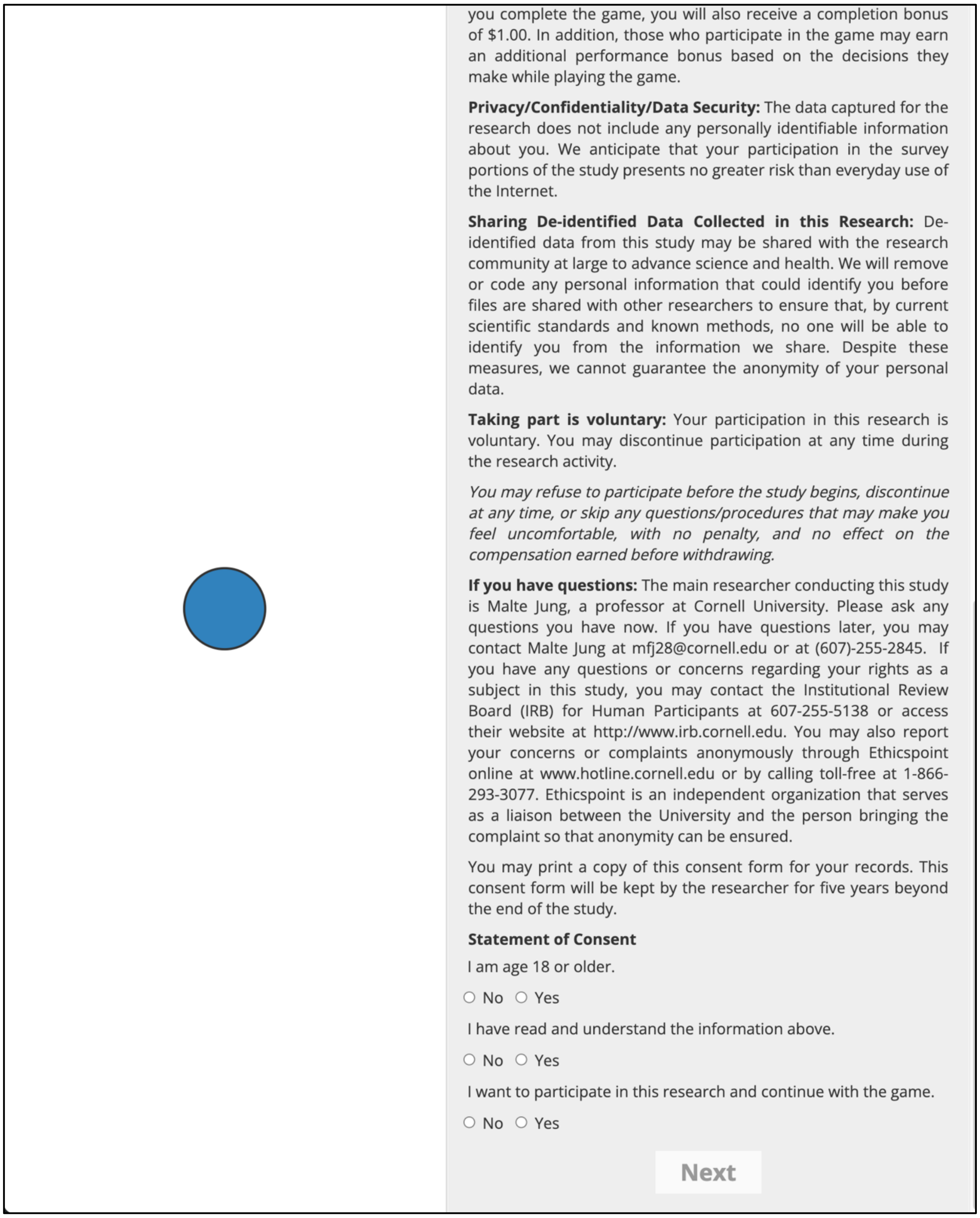
you complete the game, you will also receive a completion bonus of $1.00. In addition, those who participate in the game may earn an additional performance bonus based on the decisions they make while playing the game.

Privacy/Confidentiality/Data Security: The data captured for the research does not include any personally identifiable information about you. We anticipate that your participation in the survey portions of the study presents no greater risk than everyday use of the Internet.

Sharing De-identified Data Collected in this Research: De-identified data from this study may be shared with the research community at large to advance science and health. We will remove or code any personal information that could identify you before files are shared with other researchers to ensure that, by current scientific standards and known methods, no one will be able to identify you from the information we share. Despite these measures, we cannot guarantee the anonymity of your personal data.

Taking part is voluntary: Your participation in this research is voluntary. You may discontinue participation at any time during the research activity.

You may refuse to participate before the study begins, discontinue at any time, or skip any questions/procedures that may make you feel uncomfortable, with no penalty, and no effect on the compensation earned before withdrawing.

If you have questions: The main researcher conducting this study is Malte Jung, a professor at Cornell University. Please ask any questions you have now. If you have questions later, you may contact Malte Jung at mfj28@cornell.edu or at (607)-255-2845. If you have any questions or concerns regarding your rights as a subject in this study, you may contact the Institutional Review Board (IRB) for Human Participants at 607-255-5138 or access their website at http://www.irb.cornell.edu. You may also report your concerns or complaints anonymously through Ethicspoint online at www.hotline.cornell.edu or by calling toll-free at 1-866-293-3077. Ethicspoint is an independent organization that serves as a liaison between the University and the person bringing the complaint so that anonymity can be ensured.

You may print a copy of this consent form for your records. This consent form will be kept by the researcher for five years beyond the end of the study.

Statement of Consent

I am age 18 or older.

○ No ○ Yes

I have read and understand the information above.

○ No ○ Yes

I want to participate in this research and continue with the game.

○ No ○ Yes

Next

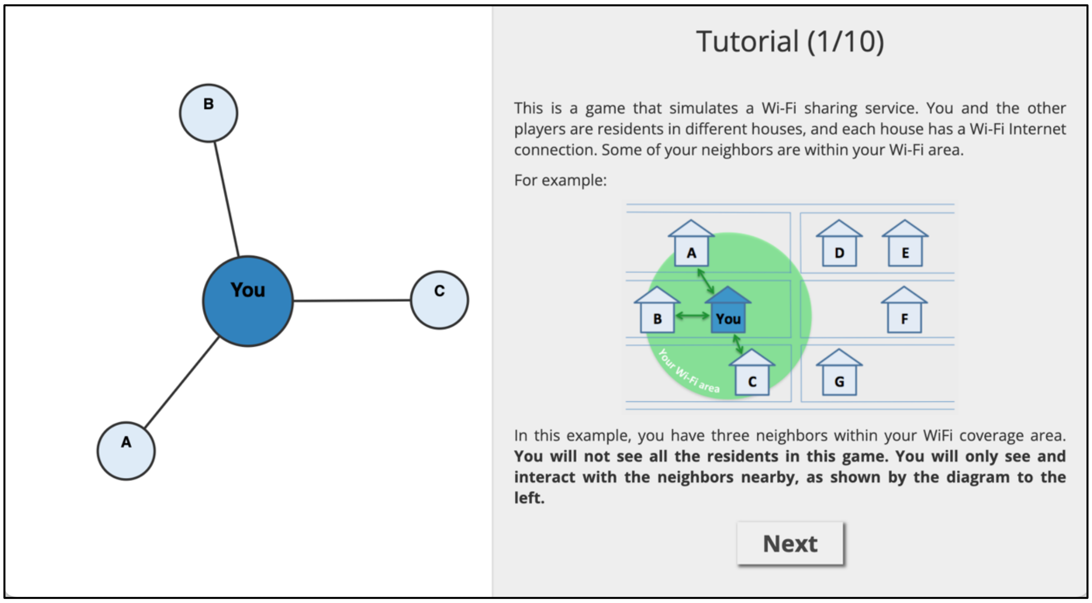
B
You
C
A
Tutorial (1/10)
This is a game that simulates a Wi-Fi sharing service. You and the other players are residents in different houses, and each house has a Wi-Fi Internet connection. Some of your neighbors are within your Wi-Fi area.
For example:
A
D
E
B
You
F
Your Wi-Fi area
C
G
In this example, you have three neighbors within your WiFi coverage area. **You will not see all the residents in this game. You will only see and interact with the neighbors nearby, as shown by the diagram to the left.**
Next

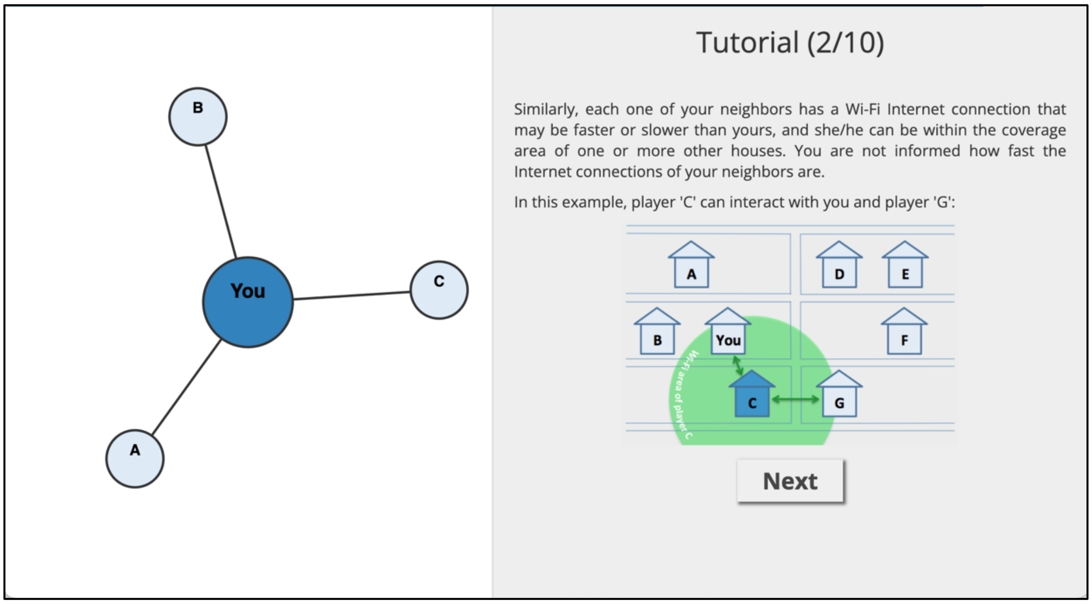
B
You
C
A
Tutorial (2/10)
Similarly, each one of your neighbors has a Wi-Fi Internet connection that may be faster or slower than yours, and she/he can be within the coverage area of one or more other houses. You are not informed how fast the Internet connections of your neighbors are.
In this example, player 'C' can interact with you and player 'G':
A
D
E
B
You
F
Wi-Fi area of player C
C
G
Next

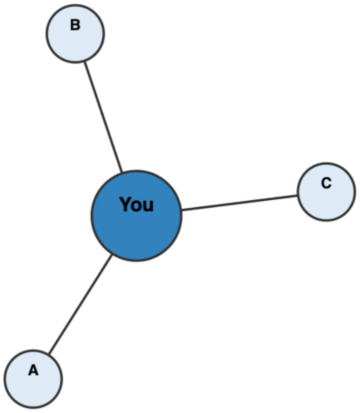

Tutorial (3/10)

The game is played in rounds and each round represents a different day. When you are not home, you are not using your Wi-Fi Internet, but you can share it with your neighbors. If your neighbors do the same, you can all enjoy a better Internet connection when you return home.

At the beginning of each round, you need to decide how much Internet bandwidth (in Mbps) you will give to each one of your neighbors. There is no cost for sharing your Internet. At the end of each round, you will find out how much Internet each of your neighbors has given to you.

When you join the game and attend it to the end, you will earn $2.0 as a base bonus. In addition, you will be paid an extra bonus of $0.005 for every 1 Mbps Internet you received from your neighbors by the end of the game. **Your own Internet will not count towards your bonus.**

Next

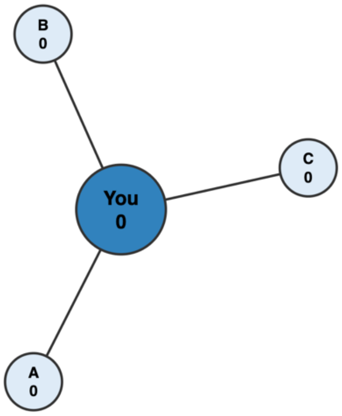

Tutorial (4/10)

Let's practice with some rounds. The practice result will not affect your bonus.

In every round, you will see the table below that shows how much of your Internet connection you shared with each one of your neighbors, and how much they shared with you in the last round. You will also see how much Internet bandwidth your neighbors have received in total (total score), both in the table and in the nodes of the left diagram.

Every morning, you can't use your Wi-fi connection because you leave home. Instead, you can share it with your neighbors.

Neighbor	Total score	You last gave	You last received	How much you give?
A	0	0	0	-
B	0	0	0	-
C	0	0	0	-
			Remaining:	-

Next

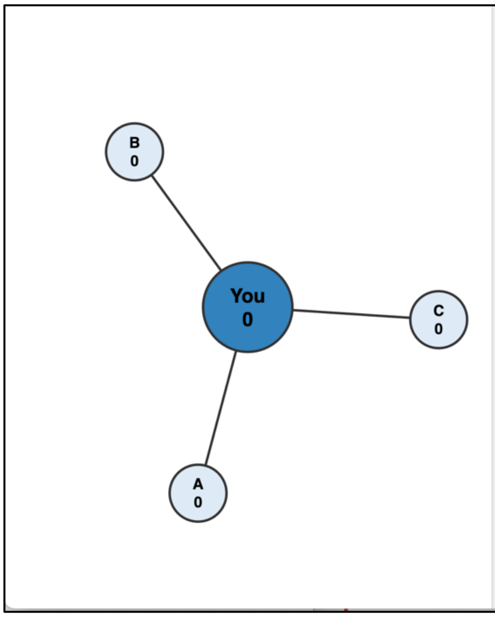

Tutorial (5/10)

Please type a number of the Internet connection (bandwidth) you want to give to each one of your neighbors. In this example, you have 30 Mbps to give away. If you don't want to give Internet to a certain neighbor, enter the number 0. You can only push the Share button after ALL the boxes have a number from 0 to 30.

Also, the sharing bandwidth cannot exceed your total bandwidth of 30 Mbps. You do not have to share all your bandwidth; for example, you can give only 20 of the 30 Mbps. However, this leftover bandwidth will not count towards your score, nor will it be available the next day.

Neighbor	Total score	You last gave	You last received	How much you give?
A	0	0	0	
B	0	0	0	
C	0	0	0	
			**Remaining:**	**30**

Share

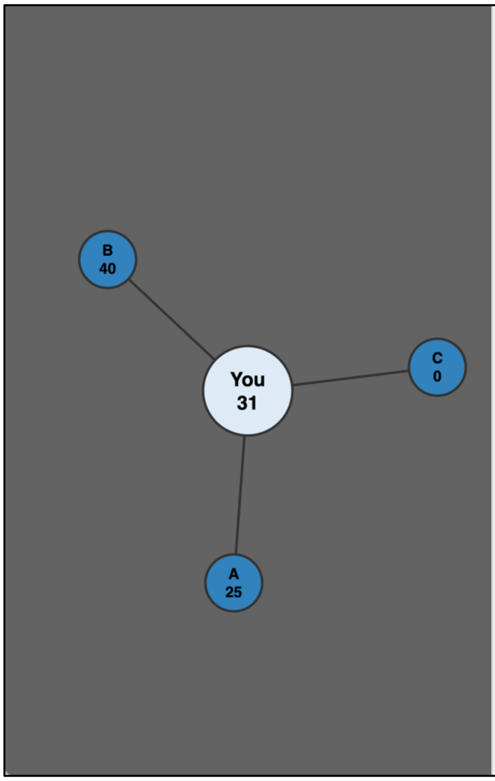

Tutorial (6/10)

In the evening, you return home and learn how much Internet connection your neighbors have given to you. The total bandwidth you received on this day is 31 Mbps. If it were the real game, this would count for your bonus at the end of this HIT ($0.005 per 1 Mbps). From now on, the total bandwidth you have received from your neighbors shows as "your total score".

You are then asked to evaluate your feeling using emojis (from very unhappy to very happy). Please select an emoji beneath the table to describe your feeling with the last result.

Your total score: 31 Mbps ($0.16)

Neighbor	Total score	You last gave	You last received	How much you give?
A	25	10	**20**	-
B	40	10	**6**	-
C	0	0	**5**	-
			Remaining:	-

How happy are you with your neighbor(s) at this moment?

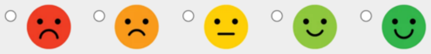

Next

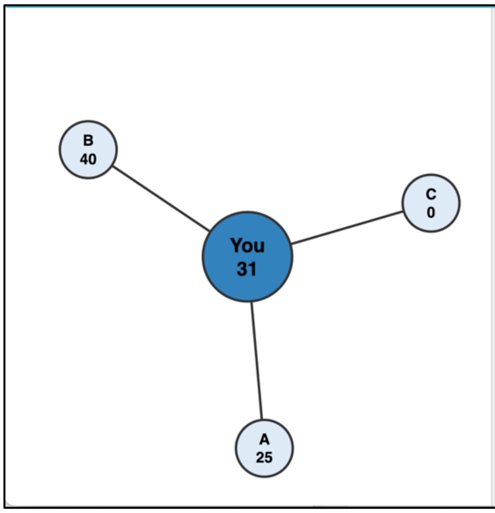

Tutorial (7/10)

The next morning (Day 2), you share your Internet connection of 30 Mbps again. You can make your Internet sharing decisions, based on your past experience.

Your total score: 31 Mbps ($0.16)

Neighbor	Total score	You last gave	You last received	How much you give?
A	25	10	20	
B	40	10	6	
C	0	0	5	
			**Remaining:**	**30**

Share

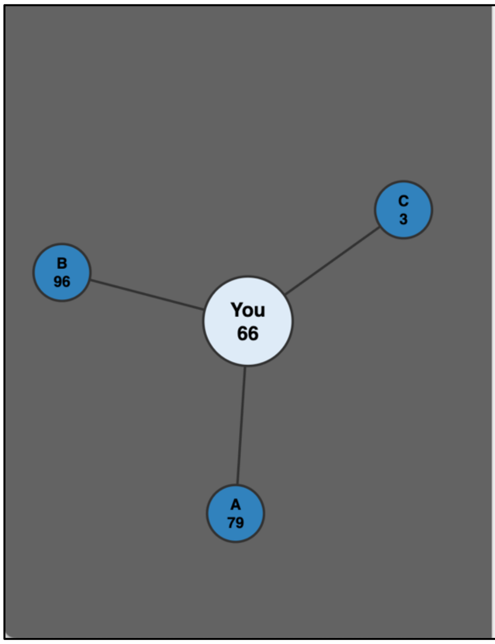

Tutorial (8/10)

In the evening (Day 2) you return home and learn how much bandwidth your neighbors have given to you. Their decisions are shown in the table below.

Your total score: 66 Mbps ($0.33)

Neighbor	Total score	You last gave	You last received	How much you give?
A	79	20	**14**	-
B	96	6	**6**	-
C	3	3	**15**	-
			Remaining:	-

How happy are you with your neighbor(s) at this moment?

Next

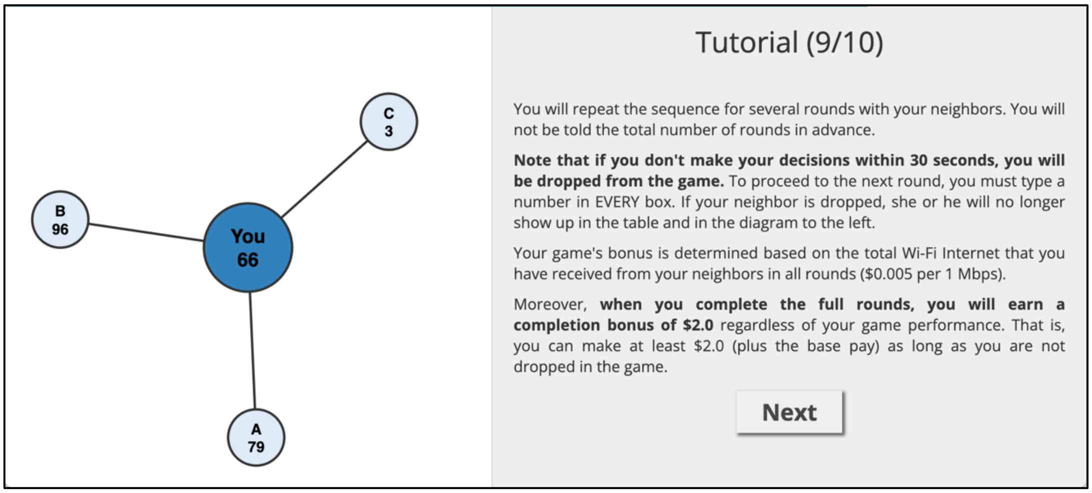
C
3
B
96
You
66
A
79
Tutorial (9/10)
You will repeat the sequence for several rounds with your neighbors. You will not be told the total number of rounds in advance.
Note that if you don't make your decisions within 30 seconds, you will be dropped from the game. To proceed to the next round, you must type a number in EVERY box. If your neighbor is dropped, she or he will no longer show up in the table and in the diagram to the left.
Your game's bonus is determined based on the total Wi-Fi Internet that you have received from your neighbors in all rounds ($0.005 per 1 Mbps).
Moreover, when you complete the full rounds, you will earn a completion bonus of $2.0 regardless of your game performance. That is, you can make at least $2.0 (plus the base pay) as long as you are not dropped in the game.
Next

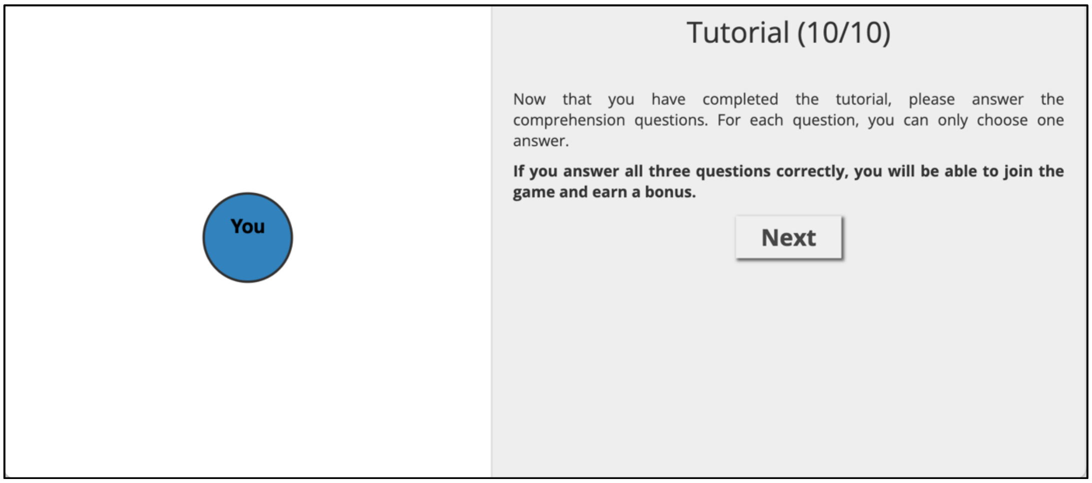
You
Tutorial (10/10)
Now that you have completed the tutorial, please answer the comprehension questions. For each question, you can only choose one answer.
If you answer all three questions correctly, you will be able to join the game and earn a bonus.
Next

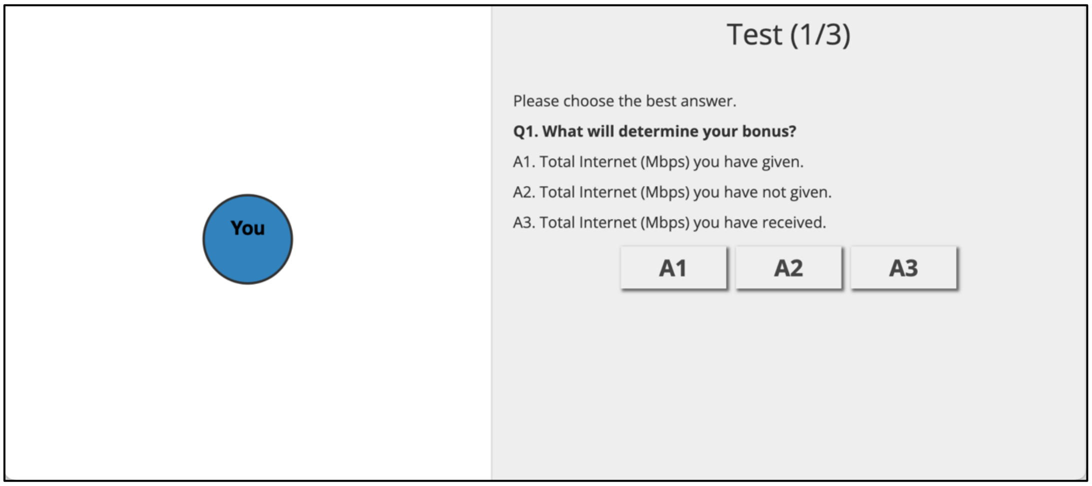
You
Test (1/3)
Please choose the best answer.
Q1. What will determine your bonus?
A1. Total Internet (Mbps) you have given.
A2. Total Internet (Mbps) you have not given.
A3. Total Internet (Mbps) you have received.
A1
A2
A3

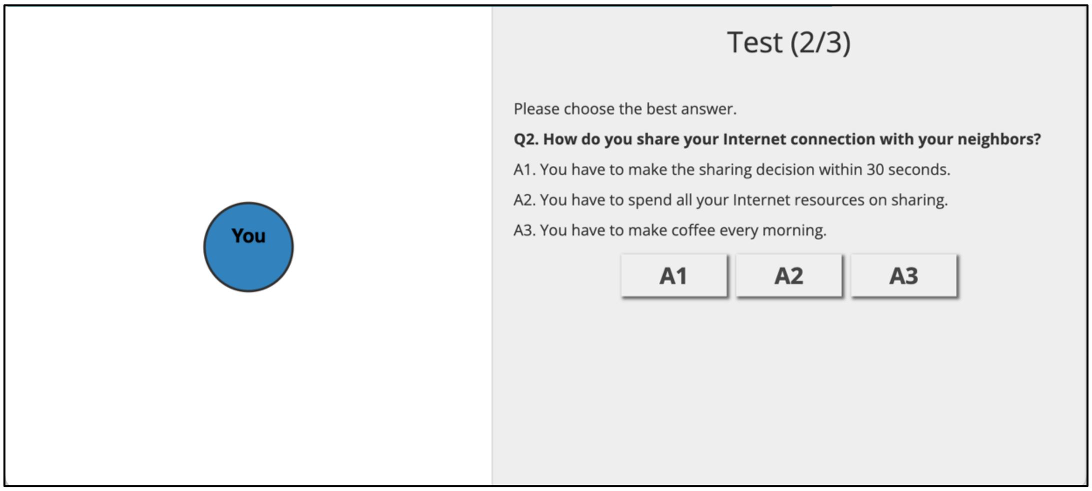
You
Test (2/3)
Please choose the best answer.
Q2. How do you share your Internet connection with your neighbors?
A1. You have to make the sharing decision within 30 seconds.
A2. You have to spend all your Internet resources on sharing.
A3. You have to make coffee every morning.
A1
A2
A3

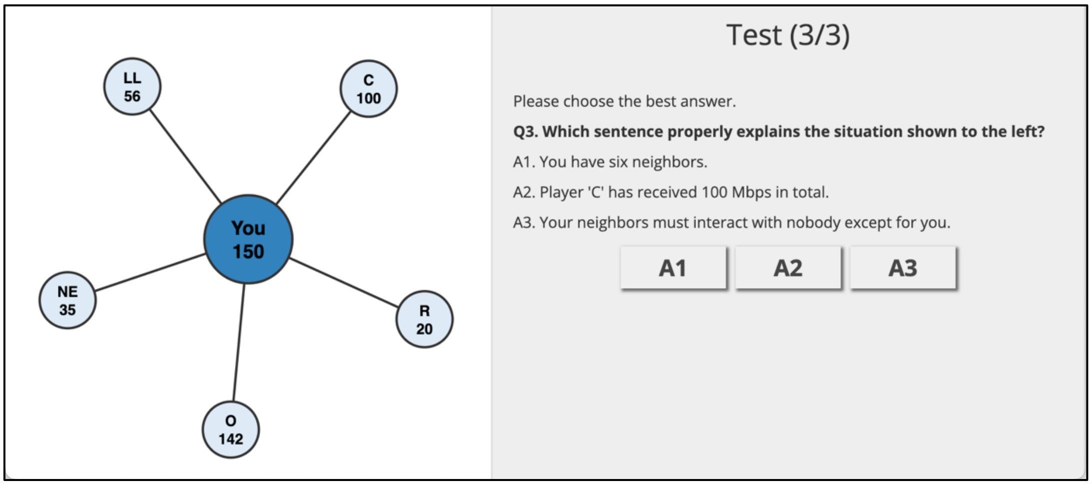
LL 56
C 100
You 150
NE 35
R 20
O 142
Test (3/3)
Please choose the best answer.
Q3. Which sentence properly explains the situation shown to the left?
A1. You have six neighbors.
A2. Player 'C' has received 100 Mbps in total.
A3. Your neighbors must interact with nobody except for you.
A1
A2
A3

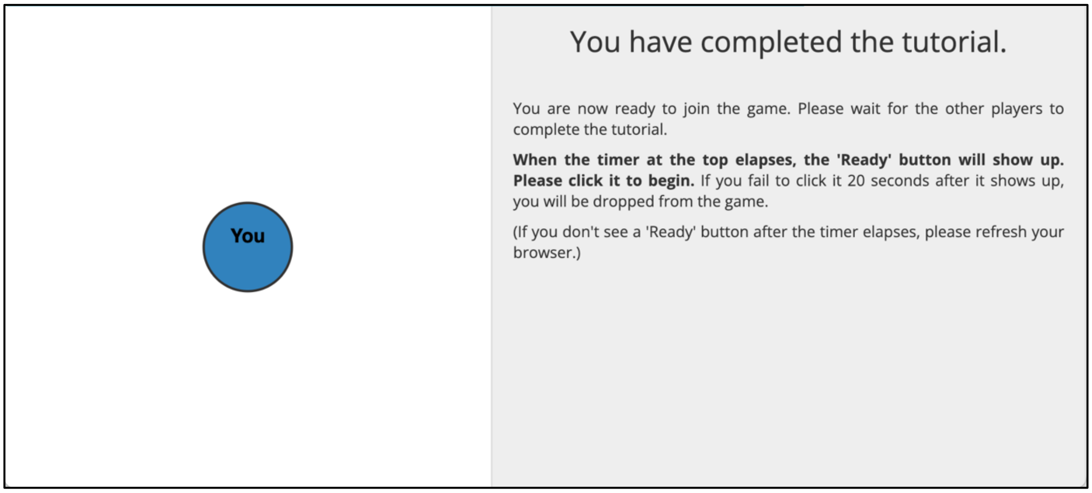
You
You have completed the tutorial.
You are now ready to join the game. Please wait for the other players to complete the tutorial.
When the timer at the top elapses, the 'Ready' button will show up. Please click it to begin. If you fail to click it 20 seconds after it shows up, you will be dropped from the game.
(If you don't see a 'Ready' button after the timer elapses, please refresh your browser.)
